# The molecular genetics of cervical carcinoma

**DOI:** 10.1038/sj.bjc.6690635

**Published:** 1999-08

**Authors:** P A Lazo

**Affiliations:** Centro de Investigación del Cáncer, Instituto de Biología Molecular y Celular del Cáncer, Consejo Superior de Investigaciones Científicas, Universidad de Salamanca, 37007 Salamanca, Spain and Unidad de Genética y Medicina Molecular, Centro Nacional de Biología Fundamental, Instituto de Salud Carlos III, Majadahonda, 28220, Spain

**Keywords:** cervical carcinoma, genetic damage, papillomavirus, viral integration, LOH

## Abstract

In the pathogenesis of cervical carcinoma there are three major components, two of them related to the role of human papillomaviruses (HPV). First, the effect of viral E6 and E7 proteins. Second, the integration of viral DNA in chromosomal regions associated with well known tumour phenotypes. Some of these viral integrations occur recurrently at specific chromosomal locations, such as 8q24 and 12q15, both harbouring HPV18 and HPV16. And third, there are other recurrent genetic alterations not linked to HPV. Recurrent losses of heterozygosity (LOH) have been detected in chromosome regions 3p14–22, 4p16, 5p15, 6p21–22, 11q23, 17p13.3 without effect on p53, 18q12–22 and 19q13, all of them suggesting the alteration of putative tumour suppressor genes not yet identified. Recurrent amplification has been mapped to 3q+ arm, with the common region in 3q24–28 in 90% of invasive carcinomas. The mutator phenotype, microsatellite instability, plays a minor role and is detected in only 7% of cervical carcinomas. The development of cervical carcinoma requires the sequential occurrence and selection of several genetic alterations. The identification of the specific genes involved, and their correlation with specific tumour properties and stages could improve the understanding and perhaps the management of cervical carcinoma. © 1999 Cancer Research Campaign


*British Journal of Cancer* (1999) **80**(12), 2008–2018
© 1999 Cancer Research Campaign
Article no. bjoc.1999.0635

### The molecular genetics of cervical carcinoma

**PA Lazo**

Centro de Investigación del Cáncer, Instituto de Biología Molecular y Celular del Cáncer, Consejo Superior de Investigaciones Científicas, Universidad de Salamanca, 37007 Salamanca, Spain and Unidad de Genética y Medicina Molecular, Centro Nacional de Biología Fundamental, Instituto de Salud Carlos III, 28220 Majadahonda, Spain

**Summary** In the pathogenesis of cervical carcinoma there are three major components, two of them related to the role of human papillomaviruses (HPV). First, the effect of viral E6 and E7 proteins. Second, the integration of viral DNA in chromosomal regions associated with well known tumour phenotypes. Some of these viral integrations occur recurrently at specific chromosomal locations, such as 8q24 and 12q15, both harbouring HPV18 and HPV16. And third, there are other recurrent genetic alterations not linked to HPV. Recurrent losses of heterozygosity (LOH) have been detected in chromosome regions 3p14–22, 4p16, 5p15, 6p21–22, 11q23, 17p13.3 without effect on p53, 18q12–22 and 19q13, all of them suggesting the alteration of putative tumour suppressor genes not yet identified. Recurrent amplification has been mapped to 3q+ arm, with the common region in 3q24–28 in 90% of invasive carcinomas. The mutator phenotype, microsatellite instability, plays a minor role and is detected in only 7% of cervical carcinomas. The development of cervical carcinoma requires the sequential occurrence and selection of several genetic alterations. The identification of the specific genes involved, and their correlation with specific tumour properties and stages could improve the understanding and perhaps the management of cervical carcinoma.

**Keywords**: cervical carcinoma; genetic damage; papillomavirus; viral integration; LOH

Cervical carcinoma (CC) is one of the most common tumours affecting women world-wide, both in incidence and mortality, with approximately 471 000 new cases diagnosed annually (Cannistra and Niloff, 1996). This tumour is associated to some specific types of human papillomaviruses (HPV), particularly types 16, 18, 33 and 42 (zur Hausen, 1994; Howley, 1995; Shah and Howley, 1995). Recent data suggest that all cases are HPV-positive (Bosch et al, 1995). The peak incidence of the disease occurs in women over 40 years of age; however, the peak incidence for HPV infection is in the 20s, therefore there is a long latency period between the time of HPV infection and cancer appearance. Despite the infection by HPV, the course of the disease has many aspects that remain unsolved (Braly, 1996; Lazo, 1988*a*). Among these issues are how a productive infection becomes silent and long-lasting, or what makes most HPV-induced cervical lesions reversible (Cannistra and Niloff, 1996), both are likely to be a consequence of the host immunological situation. And most important, what marks the transition to malignancy of the HPV-containing cell and its progression to invasive carcinoma. The course of this tumour is similar to other cancers, with the difference of having a virus as a pathogenic agent. Therefore, in cervical carcinoma, some aspects of the pathogenesis might be influenced by the viral presence. In experimental systems, the presence of a high-risk HPV is not sufficient to induce transformation and tumour progression (Chen et al, 1993). This observation was further supported by in vitro studies of HPV-containing human keratinocytes which need several chromosomal alterations in order to become immortalized and transformed (Montgomery et al, 1995; Uejima et al, 1995). But, as a cancer, CC should also have a sequential accumulation of genetic damage that is selected as the tumour phenotype progresses.

Cancer is a multigenic disease, mostly somatic (Kinzler and Vogelstein, 1996, 1998; Lengauer et al, 1998). Cancer-related genes can fall in two major categories: oncogenes that have a dominant effect and tumour suppressor genes with a recessive phenotype. Recurrent chromosomal alterations are a hallmark of most tumour types and they are markers for the presence of an oncogenesis-related gene (Rabbitts, 1994, 1997). Chromosomal translocations are the prototype of recurrent aberrant genetic damage (Mitelman et al, 1997), and their result is the activation of a gene in *cis*, the silencing of a gene, or the formation of a novel protein produced by the fusion of coding sequences located on each chromosome (Sánchez-García, 1997). The biological consequence of the translocation, by either mechanism, is either to change the proliferation or differentiation properties of the affected cell (Sánchez-García, 1997). Other recurrent changes include point mutations, gene amplifications and losses of heterozygosity (LOH). However, it is likely that the genes implicated in each type of cancer are different as individual genes, but they all may have in common belonging to the same general type of genes, reflecting the particular phenotypic aspect where they are implicated, and the stage of cancer development where they intervene, which is likely to be the reason for their selection (Tomlinson et al, 1996; Tomlinson and Bodmer, 1999).

Within the pathogenesis of cervical carcinoma, the role played by HPV might be conceptually similar to the role of a genetic predisposing mutation which will favour the appearance of the tumour in a specific organ. The subset of HPV-infected cells mimic a group of cells that have the functional equivalent of mutations in *TP53* and *RB* because of the consequence of their

*Received 30 November 1998*
*Revised 9 February 1999*
*Accepted 16 February 1999*

*Correspondence to:* PA Lazo, CNBF, Instituto de Salud Carlos III, 28220 Majadahonda (Madrid), Spain

**Table 1** Characteristics of HPV DNA integration in host genome

| |
|---|
| On viral gene structure |
| Viral DNA is linearized between L1 and L2, the size of deleted viral DNA is variable |
| Retention of regulatory region (LCR or URR) |
| Retention of E6 and E7 genes |
| Inactivation E2 transcriptional regulator |
| Loss or damage of all other viral genes: E1, L1 and L2 |
| On viral gene expression |
| E6 and E7 expression modulated by flanking cellular sequences or cellular transcription factors |
| E6 and E7 transcripts have altered message stability |
| E6 and E7 expressed from hybrid viral cellular message produce normal proteins |
| On cellular genome |
| Initial integrations might be random resulting from recombination |
| Selected integrations are near fragile sites or oncogenes |
| Integration is recurrent at some locations: 8q24 and 12q14–15 |
| Frequent deletion of cellular sequences, the rearrangement is complex |
| Altered cellular gene expression as consequence of nearby integration: *N-MYC*, *JUN-B* |

interaction with viral gene products (Banks et al, 1995; Kubbutat and Vousden, 1996). Therefore, in the pathogenesis of cervical carcinoma we can identify three major factors. Two of them are related to the HPV presence, the effects of viral E6 and E7 proteins, and the consequences of HPV DNA integration in the cellular genome. The third factor is the accumulation of cellular genetic damage, not related to HPV, needed for tumour development (Tomlinson et al, 1996; Lengauer et al, 1998).

### CONSEQUENCES OF INTEGRATION ON VIRAL GENES

Viral DNA integrated into host genome is found in all cases of cervical carcinoma (Bosch et al, 1995), their metastasis and derivative cell lines (Cullen et al, 1991). This viral DNA integration is in itself a mutation with consequences both on viral and cellular genome. Cells carrying integrated viral DNA grow better in vitro, and integration has been correlated with a poor prognosis, and development of resistance to treatment (Unger et al, 1995; Vernon et al, 1997; Kalantari et al, 1998). In the case of cervical carcinoma the integration of viral DNA can contribute to two aspects where HPV is implicated in the CC phenotype. The different consequences of HPV integration either on viral or cellular genome, reflecting the common characteristics of independent CC, are outlined in Table 1.

From the viral perspective there is only a small deletion of DNA, rarely being more than three kilobases, and retention of specific viral genes. The integrated HPV DNA is linearized between the E1 and L1 genes, in such a way that the viral regulatory region and the E6 and E7 genes are expressed from viral promoters, but with a different regulation, in which cellular factors might play an important role (Gloss et al, 1989; Rosl et al, 1991; Bartsch et al, 1992; O'Connor et al, 1996). From these hybrid viral-cellular aberrant RNA messages, normal E6 and E7 proteins are synthesized. There is also the loss, by break or deletion, of the viral E2 gene, that normally functions as a positive and/or negative regulator of transcription. Furthermore, the cellular DNA might have undergone complex rearrangements and deletions which are sometimes very large (Gallego and Lazo, 1995; Bauer-Hoffman et al, 1996; Gallego et al, 1997).

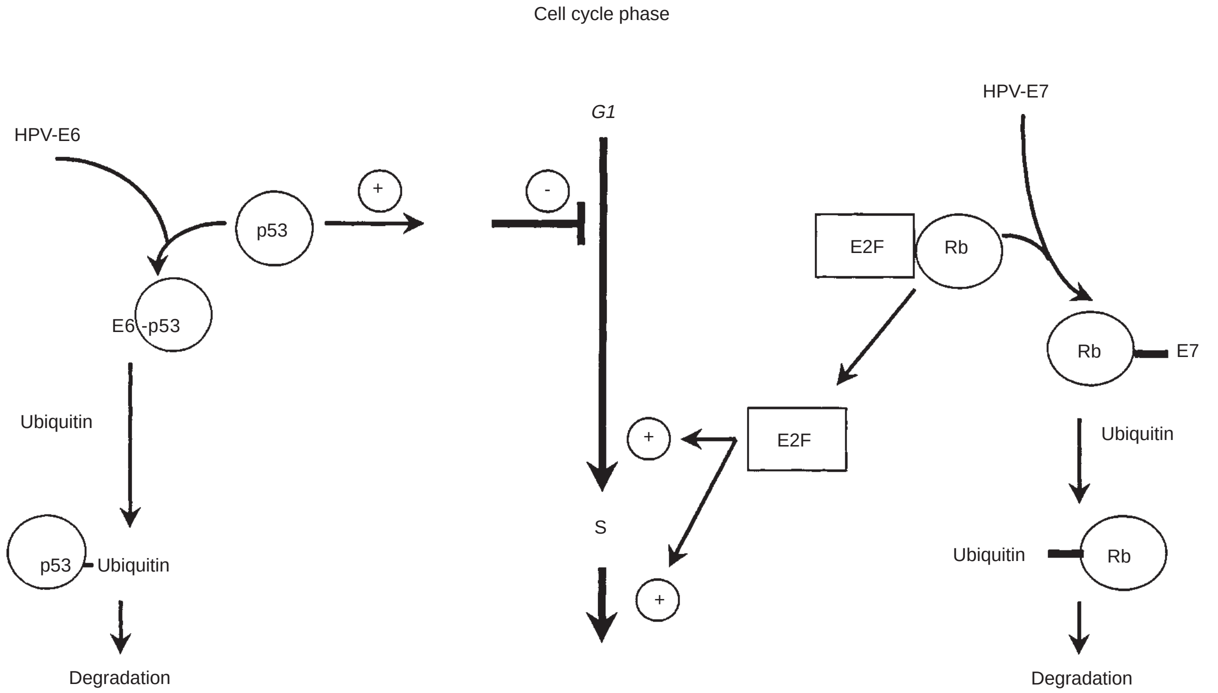

**Figure 1** Dual effect of HPV E6 and E7 proteins on the cell cycle. Possible mechanism by which HPV E6 and E7 protein can functionally mimic a state of deficiency of p53 and Rb tumour suppressor proteins. The viral proteins promote the ubiquitin-mediated degradation of both tumour suppressor proteins releasing brakes of cell cycle progression and facilitating the expression of genes also needed for completion of the cell cycle

**Table 2** Human papillomavirus chromosome integration regions and tumour phenotypes associated to these regions

| HPV type | Chromosomal region | Tumour phenotypes |
|---|---|---|
| 18 | 2p24 | Neuroblastoma, lung carcinoma |
| 16 | 2q34–35 | Sarcomas, breast adenocarcinoma, non-Hodgkin's lymphoma |
| 16 | 3p14.2 | Many types of carcinomas |
| 18 | 3p21–22 | Non-Hodgkin's lymphoma, pleomorphic adenoma, adenocarcinoma (kidney, ovary, colorectal), lung carcinoma |
| 16 | 3q25 | Ovarian carcinoma, non-Hodgkin's lymphoma |
| 18 | 5p11–15 | Adenocarcinoma (breast, kidney, lung, pancreas, stomach), AML |
| 18 | 8q22 | AML |
| 16,18 | 8q24 | Burkitt's lymphoma, ALL, malignant lymphoma, familial renal carcinoma, chondrosarcomas |
| 18 | 9q31–q34 | AML, CML, ALL |
| 16,18 | 12q14–15 | Melanoma, lipoma, liposarcoma, glioma, leiomyosarcoma, pleomorphic adenoma, T-cell lymphoma, hamartoma |
| 16 | 13q21–31 | AML, ALL, CML |
| 16 | 15q14–15 | AML |
| 16 | 19p13.2 | AML, ALL, Ewing's sarcoma, melanoma |
| 18 | 22q12–q13 abnormal | Neuroepithelioma, meningioma, Ewing's sarcoma, CML, neuroblastoma |

AML, acute myeloid leukaemia; ALL, acute lymphoblastic leukaemia; CML, chronic myeloid leukaemia

### Effects of viral E6 and E7 proteins

The effects of E6 and E7 viral genes have been extensively studied (Kubbutat and Vousden, 1996). These two viral genes are always retained and overexpressed in CC, and therefore are supposed to contribute to the tumour phenotype. The E6 and E7 proteins have been shown to induce immortalization of different cell types, such as fibroblasts (Pirisi et al, 1987) and human keratinocytes (Smith et al, 1992). In keratinocytes, immortalized with HPV and expressing E6 and E7, there is an alteration of the balance between proliferation and cell death (Smith et al, 1992), which suggests that the functions of E6 and E7 are to expand a cell population because of their effect on immortalization, probably as a consequence of their effects on apoptosis (Thomas et al, 1996; Iglesias et al, 1998; Stoppler et al, 1998). In order for these proteins to induce cellular transformation, by in vitro parameters, they require the cooperation of other genes (Kubbutat and Vousden, 1996), such as the co-transfection with the H-*ras* oncogene (Schneider et al, 1991).

The E6 protein interacts with the p53 tumour suppressor protein (Werness et al, 1990) theoretically resulting in the functional sequestration of p53 (Figure 1) and targeting it for degradation via the ubiquitin-dependent proteolytic pathway (Thomas et al, 1996). The binding of E6 to p53 might thus contribute to accumulate additional mutations by allowing progression through the cell cycle before DNA has been repaired, by altering the control checkpoint for DNA integrity in the G1 phase before entering the S phase of the cell cycle. An additional consequence could be the inhibition of apoptosis by the partial effect of p53 levels and of pRb.

The HPV E7 protein interacts with either pRb and the p107- or p130-related proteins (Figure 1). This interaction dissociates these proteins from the transcription factor E2F. The free E2F factor can activate transcription of genes that will promote cell cycle progression and cell proliferation (Kubbutat and Vousden, 1996). E7 can also interact with cellular transcription factors such as AP-1 (Antinore et al, 1996).

It is important to take into account that high levels of E6 and E7 expression can also be achieved from viral DNA in its extrachromosomal form, therefore in CC there must be additional factors that contribute to the final tumour phenotype and which might be selected, consistent with the long time that occurs between initial infection and tumour appearance. Curiously, benign tumours induced by other HPV types have a higher cell number and shorter tumour formation time than the malignant tumours. Furthermore, other oncogenic viruses, such as adenovirus and SV40, have proteins which interact with p53 and pRb even more efficiently than HPV proteins, yet they do not induce tumours in humans. Therefore, the effects of E6 and E7 may be mediated by a mechanism not yet identified.

### Inactivation of viral E2 gene by integration

The viral E2 gene codes for two proteins with a transcriptional regulatory function. The E2 protein has a transcriptional activation domain in the N-terminus and a DNA binding domain in its C-terminus. The E2 gene can be expressed in two forms, a complete protein that is a positive regulator, and a protein with only the DNA binding domain that functions as a repressor (Dowhanick et al, 1995). The full-length E2 protein induces cell cycle arrest in the S phase, thus allowing the replication of viral DNA, but at the same time resulting in an increase in cellular DNA which might be implicated in the generation of the aneuploidy observed in CC (Dowhanick et al, 1995; Frattini et al, 1997). Thus the normal E2 increases the viral load of the infected cell (early lesions) and might alter its chromosome number. In integrated viral DNA there is a loss of both types of E2 proteins. After

*© 1999 Cancer Research Campaign*

integration, the removal of this brake, will allow the expansion of cells carrying genetic anomalies of different types. The loss of E2 confers better growth properties to the cell (Jeon and Lambert, 1995; Jeon et al, 1995). The clinical relevance of the inactivation of the E2 gene was demonstrated by the analysis of 46 patients with HPV DNA integrated and with disruption of the E2 viral gene. This E2 damage was associated with poor prognosis and a significantly shortened disease-free survival of the patient (Unger et al, 1995; Vernon et al, 1997; Kalantari et al, 1998).

Additional evidence for a contributing effect of the E2 loss of function comes from different studies. In cells transfected with HPV16, the viral DNA was integrated in the genome of the host cell, and these cells acquired a growth advantage that could not be explained by the known effects of other retained viral genes, E6 and E7 (Jeon et al, 1995). Later on, it was demonstrated that the reintroduction of a normal E2 protein, from HPV16 or HPV18, in HeLa cells, suppressed the cell growth of these cells (Dowhanick et al, 1995). For this effect to take place, both the transactivating and DNA binding domains of this E2 protein have to be intact (Dowhanick et al, 1995).

### Lack of homology between viral and cellular DNA at integration sites and location of HPV DNA integration sites

One of the characteristics of cancer is the presence of recurrent chromosomal alterations associated to a specific type of tumour. These usually take the form of translocations, but also include amplifications, deletions and point mutations. These alterations are rather well characterized in human lymphoid tumours. Whereas in human solid tumours, their characterization lags behind because of the characteristics of the starting material, this has not been an obstacle for the detection of such recurrent chromosomal alteration in most types of cancer (Mitelman et al, 1997). In this context we can consider the integration of viral DNA in the cellular genome as a type of recurrent alteration in cancers associated to HPV, CC being the most extensively studied.

Viral DNA integration is irreversible genetic damage that could affect cellular genes. Several integration sites and their corresponding normal target sequences have been cloned and studied (El Awady et al, 1987; Lazo, 1988*b*; Choo et al, 1990; Gallego et al, 1997). Sequence comparisons do not detect any significant homology either between viral and target cellular DNA, or among the different cellular target regions. This lack of homology suggests that it is the result of non-homologous recombination between cellular and viral DNA. It is likely that there might be some preference for chromatin regions where cellular DNA might be more accessible (Popescu et al, 1990). In this context it is important to note that several integration regions are in chromosomal regions identified as fragile sites (Table 2) (Durst et al, 1987; Popescu et al, 1990; Lazo et al, 1992; Zimonjic et al, 1994). Direct evidence for integration within a known fragile site has recently been reported for the integration of HPV16 at 3p14.2 (Wilke et al, 1996), where FRA3B is located, within the FHIT gene (Zimonjic et al, 1997). In a population of infected cells that individually might have viral DNA integrated in different chromosome regions, those that have integrated the viral DNA in a region that confers a specific phenotype could be selected, with the final result of a non-random pattern from the point of view of the tumour. If that is the case, integration sites in tumour samples should have the integrated viral DNA in regions where there are genes or genetic alterations which most likely have already been associated to some tumour phenotype properties because this phenotype, despite individual tumour differences, is common in general terms to many tumour types.

The locations of mapped HPV integration sites in cervical carcinoma are shown in Table 2. All coincide with chromosome regions already linked to the tumour phenotype, mainly by translocations. It is worth noting that, despite the small number of cases studied, two chromosomal regions, 8q24 (Durst et al, 1987; Lazo, 1988*b*; Couturier et al, 1991) and 12q14–15 (Sastre-Garau et al, 1995; Lopez-Borges et al, 1998), are recurrently affected by both HPV-16 and HPV-18 DNA integrations. Furthermore, some genes have been shown to be directly affected by integrated HPV DNA, such as *FHIT* in 3p14–21 (Wilke et al, 1996), *MYC* in 8q24 (Durst et al, 1987; Lazo, 1988*b*; Couturier et al, 1991) and *JUN-B* in chromosome 19p13.2 (Choo et al, 1995).

### RECURRENT CELLULAR GENETIC ALTERATIONS IN CC

Cervical lesions must accumulate an increasing number of mutations as they progress towards malignancy and invasion. Therefore, the identification of recurrent chromosomal alterations is of utmost importance for the understanding of the biology of this cancer. The genetic alterations in CC might be either a consequence of biological selection for specific chromosome locations containing integrated HPV DNA or result from other alterations that are recurrently affected in this tumour. The recurrent genetic alterations might take several forms such as translocations, point mutations, amplifications, viral DNA integrations, mutator phenotypes or LOH. The LOH means that a particular DNA region has been lost, and therefore, if this loss is recurrent in a significant number of cases of a particular tumour type we can conclude that this chromosome region plays a role in that tumour, and is selected because of it (Tomlinson et al, 1996; Tomlinson and Bodmer, 1999). LOH is generally thought of as an intermediate step in the inactivation of tumour suppressor genes, such as *p53* or *RB*, which requires the inactivation of both alleles in order to display their phenotype. However, in most cases, the LOH reflects a hemizygous situation and its effect might be a dose effect rather than inactivation of a tumour suppressor gene, although both share a common mechanism of inactivation, and hence of detection, of the loss of genetic material. A particular situation might be represented by the possible existence of different alleles of tumour susceptibility gene, if one of the alleles is more effective than others, there might be a loss of the less effective allele, thus dominating the predisposing allele in these tumours.

LOH analysis has been extensively applied to CC leading to the identification of several chromosomal regions that are recurrently affected in this tumour (Table 3).

### LOH at 3p and the FHIT gene in CC

The 3p region has been implicated in ovarian cancer, breast cancer, testicular cancer, lung cancer and renal cell carcinoma, strongly suggesting the presence of a tumour suppressor gene in the region. In CC, a broad region, 3p12–24, was identified as a target for LOH. The combined data show that LOH occurs at an average frequency of 48% (Table 3), although it varies from study to study depending on the number of markers used (Jones and Nakamura, 1992; Kohno et al, 1993; Karlsen et al, 1994; Mitra et al, 1994*a*;

© 1999 Cancer Research Campaign

**Table 3** Recurrent LOH in cervical carcinomas

| Chromosome region | No. of cases (positive/informative) | % | References |
|---|---|---|---|
| 3p14.1–p22 | 186/391 | 48 | Yokota et al, 1989; Jones and Nakamura, 1992; Kohno et al, 1993; Karlsen et al, 1994; Mitra et al, 1994*a*; Mullokandov et al, 1996; Ku et al, 1997; Larson et al, 1997; Chu et al, 1998; Huettner et al, 1998; Kersemaekers et al, 1998*b* |
| 4p16 | 27/66 | 40 | Hampton et al, 1996; Mullokandov et al, 1996; Kersemaekers et al, 1998*b* |
| 4q21–35 | 23/72 | 32 | Mitra et al, 1994*a*; Mullokandov et al, 1996; Kersemaekers et al, 1998*b* |
| 5p13–15 | 20/117 | 17 | Ku et al, 1977; Mitra et al, 1994*a*; Mullokandov et al, 1996; Kersemaekers et al, 1998*b* |
| 6p21.3–22 | 60/147 | 41 | Mitra et al, 1994*a*; Mullokandov et al, 1996; Huettner et al, 1998; Kersemaekers et al, 1998*b* |
| 11p15 | 12/43 | 28 | Mitra et al, 1994*a*; Mullokandov et al, 1996 |
| 11q23 | 73/188 | 38 | Hampton et al, 1994; Bethwaite et al, 1995; Mullokandov et al, 1996; Huettner et al, 1998; Kersemaekers et al, 1998*a*, 1998*b* |
| 17p13.3 | 50/208 | 24 | Fujita et al, 1992; Kaelbing et al, 1992; Mitra et al, 1994*a*; Park et al, 1995; Mullokandov et al, 1996; Ku et al, 1997; Kersemaekers et al, 1998*b* |
| 18q12.2–22 | 18/75 | 24 | Mitra et al, 1994*a*; Mullokandov et al, 1996; Kersemaekers et al, 1998*b* |

Mullokandov et al, 1996; Ku et al, 1997; Larson et al, 1997; Chu et al, 1998; Huettner et al, 1998). These LOH were detected in both squamous cell carcinomas and adenocarcinomas. In some studies the frequency appears to be higher in squamous cell carcinomas (68%) than in adenocarcinomas (42%) (Larson et al, 1997). LOH in 3p might be an indicator of tumour progression (Table 2), since its frequency increases from 25% in FIGO stage I cases to 100% in stage IV cases (Larson et al, 1997). Among the genes from this chromosome region ruled out to be affected by LOH are *APEH*, *D8*, *GNA12B*, *ZNF*35, *RARB*, *THRB* and *RAF1* (Kohno et al, 1993).

Several studies agree that within the region 3p14–p22 there are two subregions where the LOH are concentrated, and frequently both are altered in the same tumour. These two regions are 3p14.2 and 3p21 (Kohno et al, 1993; Karlsen et al, 1994; Mitra et al, 1994*a*; Larson et al, 1997; Chu et al, 1998). These data suggest that two tumour suppressor genes are likely to be located in this chromosome region (Huettner et al, 1998; Kersemaekers et al, 1998*a*).

Recently the *FHIT* gene has been identified within the chromosome region 3p14.2, which is altered in several types of tumours, such as lung (Sozzi et al, 1996), breast (Hayashi et al, 1997) and oesophageal carcinomas (Zou et al, 1997), but not in others, such as colon carcinoma (Thiagalingam et al, 1996). These alterations are detected at the cDNA level in the form of exon losses. The *FHIT* gene spans more than 1 Mb and comprises the *FRA3B* fragile site (Zimonjic et al, 1997). It is important to note that integration of HPV16 occurred directly at the FRA3B fragile site when using HPV integration as a marker for potential oncogenic loci in HPV16 immortalized keratinocytes (Wilke et al, 1996). This integration occurred between exons 4 and 5 (Zimonjic et al, 1997). The *FHIT* gene has been specifically studied in cervical carcinomas (Greenspan et al, 1997; Chu et al, 1998; Muller et al, 1998; Yoshino et al, 1998). Aberrant FHIT transcripts were detected in seven out of 11 (63%) CC cell lines and in 35 out of 74 (47%) primary cervical carcinomas of different types and stages (Muller et al, 1998). The aberrant transcripts usually are coexpressed together with a normal transcript. This loss of normal transcript was correlated with a significant loss of FHIT protein by inmmunohistochemistry. In 25 out of 33 primary tumours there was no detectable FHIT protein, while high levels were detected in normal non-neoplastic squamous and glandular cervical epithelium (Greenspan et al, 1997). In another study of 57 cases, aberrant FHIT mRNA was detected in 9/29 (31%) of the cases, but in normal tissues similar aberrant transcripts were detected in 12/31 of the cases. Therefore it appears that *FHIT* exon skipping may not be a specific property of the tumour cell (Chu et al, 1998). The *FHIT* gene is not a typical tumour suppressor gene (Le Beau et al, 1998), and the mechanism by which it might contribute to the tumour phenotype is not known (Mao et al, 1996).

In the 3p21 region the affected gene is not known. This region has the *hMLH1* gene, responsible for a mutator phenotype. This gene does not show LOH and is unlikely to be mutated in CC because the frequency of the mutator phenotype in CC is one order of magnitude lower than the LOH frequency. Another candidate gene in the region is β-catenin, but its implication in CC has also been ruled out (Kersemaekers et al, 1998*a*). LOH at 3p21 appears to correlate with high mitotic activity (Kersemaekers et al, 1998*a*).

The importance of chromosome 3 region in CC is consistent with previous studies in experimental systems. Human

*© 1999 Cancer Research Campaign*

keratinocytes immortalized with HPV-16 DNA show LOH at 3p. The tumorigenicity of these cells in nude animals correlated very well with instability and loss of genetic material from human chromosome 3p (Montgomery et al, 1995; Steenbergen et al, 1996), 10p, 11q and 18q regions (Montgomery et al, 1995; Steenbergen et al, 1995, 1996). If these regions are the same as those showing LOH in primary tumour samples, they might be very useful for the identification of the specific genes involved. Moreover, LOH in 3p13–22 was also detected in ten out of 16 (62%) CC cell lines, which is consistent with the data obtained from primary tumours (Larson et al, 1997).

### Chromosome 4 in CC

There is experimental evidence suggesting that human chromosome 4 might be important in CC. The introduction of chromosome 4 into HeLa cells conferred a senescence phenotype (Ning et al, 1991). Furthermore, the most common chromosome aberration is the detection of an isochromosome, i(4), in CC (Atkin et al, 1989). Later, LOH studies identified at least two regions that are frequently involved in this tumour (Table 3). One of them, in the short arm, 4p16, is consistent in all studies. The second, in the long arm, has been mapped to a larger area, 4q21–35, depending on the markers used. But the general conclusion is that two genes on this chromosome contribute to the cervical carcinoma phenotype (Hampton et al, 1996; Kersemaekers et al, 1998*b*).

### The short arm of chromosome 5 in CC

The first evidence indicating that chromosome 5 might be implicated in CC was detected in the HeLa cell line. This cell line has an isochromosome 5p with integrated HPV18 (Popescu et al, 1987). Furthermore, the cytogenetic analysis of 43 cervical carcinomas, stages IIb–IV, also detected the presence of i(5p) in 75% of the cases, and frequently there were two copies of this isochromosome (Atkin et al, 1989). Later, using comparative genome hybridization (CGH), amplification of 5p was detected in a large number of cases, particularly in advanced stages, from IIb to IV (Heselmeyer et al, 1997), consistent with the cytogenetic evidence. All these data suggest a dose effect of a gene located on 5p. However, LOH studies on this chromosome have revealed a low number of affected loci (Mullokandov et al, 1996; Kersemaekers et al, 1998*b*).

### Chromosome 6 in CC

Several studies have identified a very high incidence (41%) of LOH in the short arm of chromosome 6p21.3–p25 (Mitra et al, 1994*a*; Mullokandov et al, 1996; Huettner et al, 1998; Kersemaekers et al, 1998*b*). Some groups identify two independent regions with LOH, 6p21.3 and 6p24 (Kersemaekers et al, 1998*b*). Among the candidate altered genes is *TNF-α* on 6p21.3. Using markers for this gene, LOH was detected in 17 out of 43 (40%) cases of FIGO stages I and II lesions (Kersemaekers et al, 1998*b*). Although there is still no functional evidence for the role of TNF-α in cervical carcinoma, its alteration is likely to make tumour cells less sensitive to the induction of apoptosis and therefore promote their survival (Hueber et al, 1997; Krammer, 1997).

### Chromosome 11 in CC

Three lines of work support the importance of human chromosome 11 in cervical cancer, all of them pointing to the presence of a tumour suppressor gene that plays a fundamental role in this tumour phenotype. The initial evidence came from somatic cell genetics. The fusion of HeLa cells (with integrated HPV18) or SiHa cells (with integrated HPV16), with microcells containing human chromosome 11, resulted in the generation of cell hybrids that have lost their tumorigenic properties in nude mice, but if these hybrids lost the introduced chromosome 11 they regained their tumorigenic phenotype (Saxon et al, 1986; Koi et al, 1989; Oshimura et al, 1990). Furthermore, the progression towards immortalization of human keratinocytes transfected with either HPV16 or HPV18 was accompanied by allele losses at 11q (Steenbergen et al, 1996), and cytogenetic studies of cervical carcinomas detected that chromosome 11 is underrepresented (Southern and Herrington, 1997).

Two different regions with LOH have been identified in chromosome 11, one on each arm (Srivasan et al, 1991; Hampton et al, 1994; Mitra et al, 1994a; Bethwaite et al, 1995; Mullokandov et al, 1996; Kersemaekers et al, 1998*a*, 1998*b*). Two groups detected LOH in 12 out of 43 cases (28%) in the 11p15 region, but could not show that the Wilms' tumour (*WT1*) suppressor gene is implicated in these carcinomas (Mitra et al, 1994*a*; Mullokandov et al, 1996). Other groups detected LOH in 11q22–24 (Table 3). The region where this putative tumour suppressor gene might be was narrowed down to 11q23 (Hampton et al, 1994; Bethwaite et al, 1995; Mullokandov et al, 1996; Kersemaekers et al, 1998*a*). LOH at 11q23 has a strong correlation with the presence of an invasive carcinoma at FIGO stages I and II (Kersemaekers et al, 1998*b*). However, it is not known if any of these two regions are the ones that revert the tumorigenic phenotype of HeLa and SiHa cells.

### LOH in chromosome 17 and lack of mutations in p53

Chromosome 17 was the first to attract attention in cervical carcinoma because it contains the *TP53* gene in its p13.3 band, which is frequently mutated in many types of tumours (Greeblatt et al, 1994). Therefore, several studies have attempted to determine if this chromosome region is altered. The frequency of LOH at 17p13.3 is 24% (Table 3), and most often did not affect the *TP53* locus. Thus, suggesting the candidate tumour suppressor gene in this region must be different from *TP53* (Park et al, 1995), evidence for this new gene has been recently found in lung cancer (Konishi et al, 1998). Several studies have also attempted to correlate *TP53* mutations with the HPV status of the tumour. p53 appears to be more frequently mutated in HPV-negative tumours, but this negativity has been questioned since nowadays all tumours are considered HPV-positive (Bosch et al, 1995). The frequency of mutation is rather low, less than 10%, in CC (Fujita et al, 1992; Castren et al, 1998; Helland et al, 1998*a*) when compared to mutations in other tumours, such as lung or colon carcinomas (Greeblatt et al, 1994). This lack of mutations has lent support to the interpretation that mechanism implicated in these tumours is likely to be a consequence of the interaction of HPV E6 viral protein with p53 protein (Figure 1), and by which they could achieve a biological situation resembling a partial p53 defect.

Overexpression of p53 may be found in cases of CC negative for HPV (Helland et al, 1998*a*). The analysis of the expression of p53 protein in cervical lesions detected a high level of p53 expression in low-grade SIL lesions and in lesions with HPV 6/11/42 (86%) (Kurvinen et al, 1996). But in high-grade SIL or HPV16/18-positive lesions the level of p53 was absent or detected

© 1999 Cancer Research Campaign in only a few cells (Kurvinen et al, 1996). These authors found no correlation of lesion with mdm2 protein antigen level. In the same study the bcl2 protein was confined to basal cells in normal epithelium, and in hSIL lesions positive for HPV16/18 bcl2 appeared located on suprabasal cells (Kurvinen et al, 1996).

### Chromosome 18 and 19 recurrent LOH in CC

In two other chromosomes, 18 and 19, LOH have been detected in two different regions, although these chromosomes have been studied in less detail. In chromosome 18 LOH within 18q21 region was detected in 24% of the cases (Table 3), and in a smaller proportion in 18p11 band, 22 out of 172 cases (12%) (Mitra et al, 1994*a*; Mullokandov et al, 1996; Huettner et al, 1998; Kersemaekers et al, 1998*b*). There is no information regarding the possible genes affected.

On chromosome 19 there are also two LOH regions, although their frequency is lower than 20%. LOH has been detected in 19q12–13 (Mullokandov et al, 1996; Huettner et al, 1998; Kersemaekers et al, 1998*b*) and 19p13 (Mullokandov et al, 1996; Huettner et al, 1998; Kersemaekers et al, 1998b), which are the breakpoints of the two chromosome 19 regions frequently involved in chromosomal translocations (Mitelman et al, 1997).

### Recurrent point mutations in CC

There have been several studies attempting to detect mutations in genes well known to have point mutations in other tumours, including *H-RAS*, *TP53*, *p16INK4A*, *p15INK4B RB* and other cell cycle genes. These were tested in both HPV-positive and -negative cells. Some mutations have been detected in *H-RAS* (Dokianakis et al, 1998; Leis et al, 1998). The *p15* and *p16* genes, both on chromosome 9p21 and separated by 25 kilobases, do not present mutations in CC (Kim et al, 1998). However, it is not known if the *p19ARF* gene, which shares exons with *p16* and which appears to be the cause of the phenotype previously attributed to *p16*, is affected (Kamijo et al, 1997). The data on *TP53* are dealt with in the section on chromosome 17.

### Recurrent amplifications and chromosome gain

Gene amplification and chromosome gain are two different mechanisms by which a tumour cell can increase a gene dosage. In a study of 22 protooncogenes in 50 primary untreated squamous cell carcinomas of the uterine cervix, clinical stages II and III, only 12 (24%) have some amplifications (Mitra et al, 1994*a*, 1994*b*). Fivefold or larger amplifications were found for *MYCL1*, *SEA*, *CCND1*, *BCL1* and *GLI* genes in one case (2%); *HRAS* was amplified in two cases (4%); and *ERBB2* in seven cases (14%). *SEA*, *CCND1* and *BCL1* map to band 11q13 and might be within a unique amplicon. Two of the *ERBB2* cases also showed rearrangement of the 17q11.2–12 band, suggesting a possible damage of this gene. Overexpression of *ERBB2* gene has been previously reported in 60% of the cases studied (Pinion et al, 1991). ERB2/neu amplification has been observed in other carcinomas, including breast and ovary (Mitra et al, 1994*b*). The protooncogenes that have been studied and that showed no amplification were: *NMYC*, *RAF1*, *KIT*, *ROS1*, *EGFR*, *MDR1*, *MET*, *MYC*, *INT2*, *KRAS2*, *ETS1*, *WNTI*, *MDM2*, *SRC* and *PDGF*.

The uterine cervix has been used to study chromosomal aberrations during the transition from premalignant to invasive carcinoma (Heselmeyer et al, 1996). CC has well-defined progression steps based on cytology that are useful for correlation with genetic aberrations. These authors studied normal cervical epithelium, mild, moderate and severe dysplasias as well as CC. The first alteration detected when there is an increase in cell proliferation is the appearance of tetraploidy. No other recurrent aberration was observed in the transition from normal to moderate dysplasia. In one severe dysplasia a gain of 3q (one out of 13) was observed. In invasive carcinomas a gain of 3q was detected in nine out of ten cases (90%). Thus, gain of 3q can be considered a late and important alteration for the developments of invasive cervical carcinoma. The smaller common region of amplification has been reduced to band 3q24–28 (Heselmeyer et al, 1996).

### Microsatellite instability and mutator phenotype

Alterations in DNA repair genes result in the accumulation of mutations, some of which are important for tumour development. DNA repair genes belong to a family of genes designated as caretaker genes (Kinzler and Vogelstein, 1998). The phenotype of mutations in this type of genes is detected by microsatellite instability (MI). The cell with a defect in any of these genes will invariably progress to develop a cancer (Kinzler and Vogelstein, 1998). In a study of 89 primary tumours, ten cervical carcinoma cell lines and 30 loci located on 3p, 4 and 11q were analysed for MI. Three out of 89 tumours (3.3%) exhibited novel tumour-specific alleles at 77, 70 and 60% of the loci studied respectively (Larson et al, 1996). Two other tumours also have some MI using additional markers, bringing the total to 5/89 (5.6%). In this work the implication of the *hMLH1*, in 3p21, was ruled out. Also one cell line, C33A which is negative for HPV, presented this mutator phenotype, with new alleles in 30 of 84 markers determined (Larson et al, 1996). In another study, alterations at eight dinucleotide loci mapping to six different chromosomes were found in five out of 82 cervical cancers (Helland et al, 1997; Kersemaekers et al, 1998*a*). In another 51 cases, mutator phenotypes were detected in four cases (Ku J-L et al, 1977; Ku W-H et al, 1997). This instability affected to one or two alleles at one or few loci. The use of semi-automated fluorescence-based detection allowed the identification of additional five out of 58 cases showing MI (Hampton et al, 1996). A more recent study has found microsatellite instability in a larger number of cases, in nine out of 64 cases (14%) (Kersemaekers et al, 1998*a*).

Overall, of 344 cases analysed for the presence of the mutator phenotype, only 28 displayed MI (8%), independently of the type of allele and number of chromosomes studied (Ku J-L et al, 1977; Larson et al, 1996; Helland, 1997; Ku W-H et al, 1997), which represents a small subset of the total number of CC cases.

### Multiple genetic changes in CC: combined genetic alterations in individual tumours

Based on the multigenic nature of cancer it should be expected that there must be several genetic alterations in a single tumour. These alterations might have occurred with an order of selection depending on the stage where their phenotypic consequences are required for the development and progression of the carcinomas. The genetic analysis of CC in this context is rather limited. But there is enough information pointing to the multigenic nature of the process. In addition to viral DNA integration is present in all

 *© 1999 Cancer Research Campaign*

CC (Bosch et al, 1995). Two studies have detected multiple LOH in several tumours (Mitra et al, 1994*a*; Mullokandov et al, 1996). The number of LOH per tumour ranged from one to ten, with an average number of four and a standard deviation of two, for 52 cases (Mitra et al, 1994*a*). Some of the locations appear consistently in independent studies, but two of them are particularly striking, those occurring in chromosomes 3 and 11. Also, some of these LOH regions coincide with reported regions of HPV DNA integration as is the case for chromosome regions 5p, 3p and 8q. In the HeLa cell line there are alterations in four oncogenic regions, 5p11–15, 8q24, 9q31–34 and 22q12–13, surprisingly all of them with integrated viral DNA (Popescu and DiPaolo, 1989). A very recent study using CGH has detected some of these combinations of genetic alterations in different types of cervical lesions (Kirchhoff et al, 1999).

In experimental immortalization of human keratinocytes with HPV 16 and HPV 18 there is LOH at chromosome region 3p (Montgomery et al, 1995; Steenbergen et al, 1996), which is combined with losses at chromosome arms 11q, 18q or 10p (Steenbergen et al, 1996), some of which have also been detected in primary tumours.

### Other factors: genetic predisposition, the role of the HLA system

In the pathogenesis of HPV-infected cells there are several aspects that appear to be a consequence of the host immune response, such as the reversion of lesions. The study of HLA haplotypes in a case control study has demonstrated that in CIN II–III lesions certain class II haplotypes, such as DQA1*0102 and DQB1*0602, are overrepresented (Helland et al, 1998*b*). Considering the widespread HPV-infected population, this observation might mean that certain haplotypes might be more efficient than others in triggering a host response that will eliminate the HPV-infected cells. The overrepresented HLA alleles may be those that are less effective in triggering a response against the HPV-infected cell. Thus, the HPV infection that is highly prevalent starts to skew the representation of HLA alleles as the disease progresses. This might be a reflection of the host immune response to the presence of HPV. Therefore, the HLA haplotype is an important factor at the initial stage of the disease because it influences the point between reversion and progression of the HPV-induced lesion. Furthermore, in CC there is a down-regulation of class I antigens in tumour cells, thus minimizing the consequences of a possible anti-tumour cell-mediated response by the host immune system (Bartholomew et al, 1997).

The host immune response can also be influenced by the type of viral antigens that might be produced during disease progression. In the initial infection with possible production of viral particles, the main viral antigens are likely to be derived from the L1 and L2 capsid proteins. However, in CC with integrated HPV DNA, the viral L1 and L2 genes are lost, and the epitopes available will be derived from the intracellular expression of the viral E6 and E7 proteins. Thus, depending on the stage of the disease, the type of host immune response directed against HPV-containing cells is likely to be different. These differential viral protein expressions will have to be taken into account for the design of HPV vaccines. In lesions with integrated viral DNA and consequent loss of viral L1 and L2 genes, the immune response will have to be against peptides derived from the E6 and E7 viral proteins.

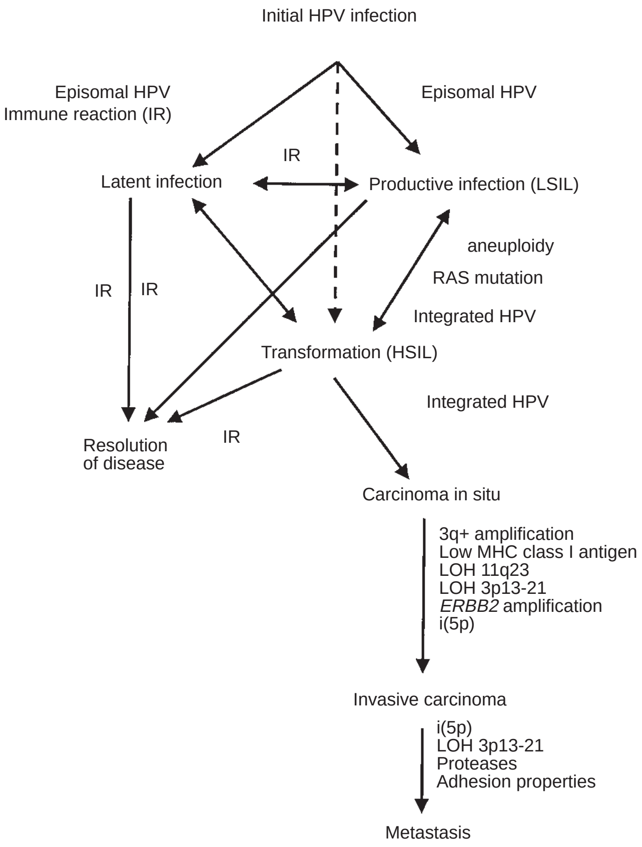

**Figure 2** Possible outcomes of HPV-infected cells. At the sequential stages of the pathogenic process of cervical carcinoma different types of specific genetic alterations are indicated. These alterations will be important for the progression of the lesion to a more advanced clinical stage

### GENETIC DAMAGE AND CERVICAL LESION STAGING

The ultimate goal of the identification of genetic damage implicated in CC would be to understand its role within the biology of this tumour phenotype and to translate this information in such a way that at diagnosis stage one could predict the evolution and outcome of the tumour depending on the mutations detected. At this moment few specific LOH have been correlated with any particular stage (Figure 2), but it is expected that in a few years, instead of LOH we will be talking about specific genes and particular tumour cell properties. Today, some of the genetic damage can be correlated with some stages along the progression of the lesion, thus indicating the stage for which their phenotypic contribution is important. Some genetic alterations such as viral DNA integration, presence of an isochromosome i(5p), 3q amplification and LOH at 3p13–21 and 11q23 are important contributors for the development of an invasive carcinoma. The timing of these genetic alterations is presented in Figure 2. The day that specific mutations can be identified routinely in a clinical setting is likely to improve the management of specific tumour types because of its potentially predictable value.

Few studies have so far addressed the possible correlation between LOH and tumour stage and evolution. For example, LOH at 11q23 appears to correlate with extensive lymphovascular space invasion, and these tumours tend to recur (Huettner et al, 1998), and tumours with LOH on 18q have a relatively poor survival (Kersemaekers et al, 1998*b*).

© 1999 Cancer Research Campaign

The next step will be to identify which gene in each chromosome region is the relevant one for cervical carcinoma, and to determine at what stage of tumour development they contribute. Thus they will become markers of specific properties related to tumour progression, and this knowledge we hope will contribute to improve management of the patients.

### ACKNOWLEDGEMENTS

The work in our laboratory was supported by grants from Fondo de Investigación Sanitaria (FIS98/0313) and Comunidad de Madrid (8.1/0004/98).

### REFERENCES

Antinore MJ, Birrer MJ, Patel D, Nader L and McCance DJ (1996) The human papillomavirus type 16 E7 gene product interacts with and trans-activates the AP1 family of transcription factor. *EMBO J* **15**: 1950–1960

Atkin NB, Baker MC and Fox MF (1989) Chromosome changes in 43 carcinomas of the cervix uteri. *Cancer Genet Cytogenet* **44**: 229–241

Banks L, Edmonds C and Vousden KH (1995) Ability of the HPV16 E7 protein to bind Rb and induce DNA synthesis is not sufficient for efficient transforming activity in NIH3T3 cells. *Oncogene* **5**: 1383–1389

Bartholomew IS, Glenville S, Sarkar S, Burt DJ, Stanley MA, Ruiz-Cabello F, Chengang J, Garrido F and Stern PL (1997) Integration of high-risk human papillomavirus DNA is linked to the down-regulation of class I antigens by steroid hormones in cervical tumor cells. *Cancer Res.* **57**: 937–942

Bartsch D, Boye B, Baust C, zur Hausen H and Schwarz E (1992) Retinoic acid-mediated repression of human papillomavirus 18 transcription and different ligand regulation of the retinoic acid receptor beta gene in non-tumorigenic and tumorigenic HeLa hybrid cells. *EMBO J* **11**: 2283–2291

Bauer-Hoffman R, Borghouts C, Bourda E, F, Rs and Alonso A (1996) Genomic cloning and characterization of the nonoccupied allele corresponding to the integration site of human papillomavirus type 16 DNA in the cervical cancer cell line SiHa. *Virology* **217**: 33–41

Bethwaite PB, Koreh J, Herrington CS and McGee JO (1995) Loss of heterozygosity occurs at the D11S29 locus on chromosome 11q23. *Br J Cancer* **71**: 814–818

Bosch FX, Manos MM, Muñoz N, Sherman M, Jansen AM, Peto J, Schiffman MH, Moreno V, Kurman R and Shah KV (1995) Prevalence of human papillomavirus in cervical cancer: a worldwide perspective. *J Natl Cancer Inst* **87**: 796–802

Braly P (1996) Preventing cervical cancer. *Nat Med* **2**: 749–751

Cannistra SA and Niloff J (1996) Cancer of the uterine cervix. *N Engl J Med* **334**: 1030–1038

Castren K, Vahakangas K, Heikkinen E and Ranki A (1998) Absence of p53 mutations in benign and pre-malignant male genital lesions with over-expressed p53 protein. *Int J Cancer* **77**: 674–678

Couturier J, Sastre-Garau X, Schneider-Maunoury S, Labib A and Orth G (1991) Integration of papillomavirus DNA near MYC genes in genital carcinomas and its consequences for proto-oncogene expression. *J Virol* **65**: 4534–4538

Cullen AP, Reid R, Campion M and AT, Lr (1991) Analysis of the physical state of different human papillomavirus DNAs in intraepithelial and invasive cervical neoplasia. *J Virol* **65**: 606–612

Chen T, Pecoraro G and Defendi V (1993) Genetic analysis of in vitro progression of human papillomavirus transfected human cervical cells. *Cancer Res* **53**: 1167–1171

Choo K, Lee H, Chong K and Chou H (1990) Analysis of the unoccupied site of an integrated human papilomavirus 16 sequence in a cervical carcinoma. *Virology* **178**: 621–625

Choo K, Huang C, Chen CM, Han CP and Au LC (1995) Jun-B oncogene aberrations in cervical cancer cell lines. *Cancer Lett* **93**: 249–253

Chu T-Y, Shen C-Y, Chiou Y-S, Perng C-L, Yu M-S and Liu H-S (1998) HPV-associated cervical cancers show frequent allelic loss at 3p14 but no apparent aberration of FHIT mRNA. *Int J Cancer* **75**: 199–204

Dokianakis DN, Sourvinos G, Sakkas S, Athanasiadou E and Spandidos DA (1998) Detection of HPV and ras gene mutations in cervical smears from female genital lesions. *Oncol Rep* **5**: 1195–1198

Dowhanick JJ, McBride AA and Howley PM (1995) Suppression of cellular proliferation by the papillomavirus E2 protein. *J Virol* **69**: 7791–7799

Durst M, Croce C, Gissman L, Schwarz E and Huebner K (1987) Papillomavirus sequences integrate near cellular oncogenes in some cervical carcinomas. *Proc Natl Acad Sci USA* **84**: 1070–1074

El Awady M, Kaplan JB, SJ, OB and Burk RD (1987) Molecular analysis of integrated human papillomavirus 16 sequences in the cervical cancer cell line SiHa. *Virology* **159**: 389–398

Frattini MG, Hurst SD, Lim HB, Swaminathan S and Laimins LA (1997) Abrogation of a mitotic checkpoint by E2 proteins from oncogenic human papillomaviruses correlates with increased turnover of the p53 tumor suppressor protein. *Embo J* **16**: 318–331

Fujita M, Inoue M, Tanizawa O, Iwamoto S and Enomoto, T (1992) Alterations of p53 gene in human primary cervical carcinoma with and without human papillomavirus infection. *Cancer Res* **52**: 5323–5328

Gallego MI and Lazo PA (1995) Deletion in human chromosome region 12q13–15 by integration of human papillomavirus DNA in a cervical carcinoma cell line. *J Biol Chem* **270**: 24321–24326

Gallego MI, Shoenmakers EPFM, Van de Ven WJM and Lazo PA (1997) Complex genomic rearrangement within the 12q15 multiple aberration region induced by integrated human papillomavirus 18 in a cervical carcinoma cell line. *Mol Carcinogen* **19**: 114–121

Gloss B, Chong T and Bernard H (1989) Numerous nuclear proteins bind the long control region of human papillomavirus type 16: a subset of 6 of 23 DNaseI-protected segments coincides with the location of the cell-type-specific enhancer. *J Virol* **63**: 1142–1152

Greeblatt MS, Bennet WP, Hollstein M and Harris CC (1994) Mutations in the p53 tumor suppressor genes: clues to cancer etiology and molecular pathogenesis. *Cancer Res* **54**: 4855–4878

Greenspan DL, Connolly DC, Wu R, Lei RY, Vogelstein JTC, Kim Y, Mok JE, Muñoz N, Bosch FX, Shah K and Cho KR (1997) Loss of FHIT expression in cervical carcinoma cell lines and primary tumors. *Cancer Res* **57**: 4692–4698

Hampton GM, Larson AA, Baergen RN, Sommers RL, Kren S and Cavanee WK (1996) Simultaneous assessment of loss of heterozygosity at multiple microsatellite loci using semi-automated fluorescence based detection: subregional mapping of chromosome 4 in cervical carcinoma. *Proc Natl Acad Sci USA* **93**: 6704–6709

Hampton GM, Penny LA, Baergen RN, Larson A, Brewer C, Liao S, Busby-Earle RMC, Williams AWR, Steel CM, Bird CC, Stanbridge EJ and Evans GA (1994) Loss of heterozygosity in cervical carcinoma: subchromosomal localization of a putative tumor-suppressor gene to chromosome 11q22–q24. *Proc Natl Acad Sci USA* **91**: 6953–6957

Hayashi S, Tanimoto K, Hajiro-Nakanishi K, Kurosomi M, Higashi Y, Imai K, Suga K and Nakachi K (1997) Abnormal FHIT transcript in human breast carcinomas: a clinicopathological and epidemiological analysis of 61 Japanese cases. *Cancer Res* **57**: 1981–1985

Helland A, Borresen-Dale A, Peltomaki P, Kristensen M, Nesland JM, De La Chapelle A and Lothe RA (1997) Microsatellite instability in cervical and endometrial carcinomas. *Int J Cancer* **70**: 499–501

Helland A, Karlsen F, Due EU, Holm R, Kristensen G and Borresen-Dale, A (1998*a*) Mutations in the TP53 gene and protein expression of p53, MDM 2 and p21/WAF-1 in primary cervical carcinomas with no or low human papillomavirus load. *Br J Cancer* **78**: 69–72

Helland A, Olsen AO, Gjoen K, Akselsen HE, Sauer T, Magnus P, Borresen-Dale AL and Ronningen KS (1998*b*) An increased risk of cervical intra-epithelial neoplasia grade II-III among human papillomavirus positive patients with the HLA-DQA1*0102–DQB1*0602 haplotype: a population-based case-control study of Norwegian women. *Int J Cancer* **76**: 19–24

Heselmeyer K, Schröck E, Du Manoir S, Blegen H, Shah KV, Steinbeck R, Auer G and Ried T (1996) Gain of chromosome 3q defines the transition from severe dysplasia to invasive carcinoma of the uterine cervix. *Proc Natl Acad Sci USA* **93**: 479–484

Heselmeyer K, Macville M, Schrock E, Blegen H, Hellstrom A-C, Shah K, Auer G and Ried T (1997) Advanced-stage cervical carcinomas are defined by a recurrent pattern of chromosomal aberrations revealing high genetic instability and a consistent gain of chromosome arm 3q. *Genes Chromosom Cancer* **19**: 233–240

Howley PM (1995) Papillomavirinae: The viruses and their replication. In *Virology*, Fields BN, Knipe DM and Howley PM (eds), Vol 2. pp. 2045–2076. Lippincot-Raven: Philadelphia

Hueber A, Zornig M, Lyon D, Suda T, Nagata S and Evan GI (1997) Requirement for the Cd95 receptor-ligand pathway in c-myc-induced apoptosis. *Science* **278**: 1305–1309

Huettner PC, Gerhard DS, Li L, Gersell DJ, Dunnigan K, Kamarasova T and Rader JS (1998) Loss of heterozygosity in clinical stage IB cervical carcinoma:

 © 1999 Cancer Research Campaign relationship with clinical and histopathologic features. *Hum Pathol* **29**: 364–370
Iglesias M, Yen K, Gaiotti D, Hildesheim A, Stoler MH and Woodsworth CD (1998) Human papillomavirus type 16 E7 protein sensitizes cervical keratinocytes to apoptosis and release of interleukin 1a. *Oncogene* **17**: 1195–1205
Jeon S and Lambert P (1995) Integration of human papillomavirus type 16 DNA in the human genome leads to increased stability of E6 and E7 mRNAs: implications for cervical carcinogenesis. *Proc Natl Acad Sci USA* **92**: 1654–1658
Jeon S, Allen-Hoffmann BL and Lambert PF (1995) Integration of human papillomavirus type 16 into the human genome correlates with a selective growth advantage of cells. *J Virol* **69**: 2989–2997
Jones MH and Nakamura Y (1992) Deletion mapping of chromosome 3p in female genital tract malignancies using microsatellite polymorphisms. *Oncogene* **7**: 1631–1634
Kaelbing M, Burk RD, Atkin NB, Johnson AB and Klinger HP (1992) Loss of heterozygosity on chromosome 17p and mutant p53 in HPV-negative cervical carcinomas. *Lancet* **340**: 140–142
Kalantari M, Karlsen F, Kristensen G, Holm R, Hagmar B and Johansson B (1998) Disruption of the E1 and E2 reading frames of HPV16 in cervical carcinoma is associated with poor prognosis. *Int J Gynecol Pathol* **17**: 146–153
Kamijo T, Zindy F, Roussel MF, Quelle DE, Downing JR, Ashmun RA, Grosveld G and Sherr CJ (1997) Tumor suppression at the INK4a locus mediated by the alternative reading frame product p19arf. *Cell* **91**: 649–659
Karlsen F, Rabbitts PH, Sundresan V and Hagmar B (1994) PCR-RFLP studies on chromosome 3p in formaldehyde-fixed paraffin-embedded cervical cancer tissues. *Int J Cancer* **58**: 787–792
Kersemaekers AM, Hermans J, Fleuren GJ and van de Vijver MJ (1998*a*) Loss of heterozygosity for defined regions on chromosomes 3, 11 and 17 in carcinomas of the uterine cervix. *Br J Cancer* **77**: 192–200
Kersemaekers AM, Kenter GG, Hermans J, Fleuren GJ and van de Vijver MJ (1998*b*) Allelic loss and prognosis in carcinoma of the uterine cervix [In Process Citation]. *Int J Cancer* **79**: 411–417
Kim JW, Namkoong SE, Ryu SW, Kim HS, Shin JW, Lee JM, Kim DH and Kim IK (1998) Absence of p15INK4B and p16INK4A gene alterations in primary cervical carcinoma tissues and cell lines with human papillomavirus infection [In Process Citation]. *Gynecol Oncol* **70**: 75–79
Kinzler KW and Vogelstein B (1996) Lessons from hereditary colorectal cancer. *Cell* **87**: 159–170
Kinzler KW and Vogelstein B (1998) Landscaping the cancer terrain. *Science* **280**: 1036–1037
Kirchhoff M, Rose H, Petersen BL, Maahr J, Gerdes T, Lundsteen C, Bryndorf T, Kryger-Baggesen N, Christensen L, Engelholm SA and Philip J (1999) Comparative genomic hybridization reveals a recurrence pattern of chromosomal aberrations in severe dysplasia/carcinoma in situ of the cervix and in advanced-stage cervical carcinoma. *Genes Chrom Cancer* **24**: 144–150
Kohno T, Takayama H, Hamaguchi M, Takano H, Yamaguchi N, Tsuda H, Hirohashi S, Vissing H, Shimizu M, Oshimura M and Yokota J (1993) Deletion mapping of chromosome 3p in human uterine cervical cancer. *Oncogene* **8**: 1825–1832
Koi M, Morita H, Yamada H, Satoh H, Barrett JC and Oshimura M (1989) Normal human chromosome 11 suppresses tumorigenicity of human cervical tumor cell line SiHa. *Mol Carcinog* **2**: 12–21
Konishi H, Takahashi T, Kozaki K, Yatabe Y, Mitsudomi T, Fujii Y, Sugiura T and Matsuda H (1998) Detailed deletion mapping suggests the involvement of a tumor suppressor gene at 17p13.3, distal to p53, in the pathogenesis of lung cancers. *Oncogene* **17**: 2095–2100
Krammer PH (1997) The tumor strikes back: new data on expression of CD95 (APO-1/FAS) receptor/ligand system may cause paradigm changes in our view on drug treatment and tumor immunology. *Cell Death Diff* **4**: 362–364
Ku J-L, Kim W-H, Park H-S, Kang S-B and Park J-G (1977) Establishment and characterization of 12 uterine cervical carcinoma cell lines: common sequence variation in the E7 gene of HPV-16 positive cell lines. *Int J Cancer* **72**: 313–320
Ku W-H, Liu I-L, Yen M-S, Chien CC, Yue C-T, Ma Y, Chang S, Ng H, Wu C and Shen C (1997) Genomic deletion and p53 inactivation in cervical carcinoma. *Int J Cancer* **72**: 270–276
Kubbutat MHG and Vousden KH (1996) Role of E6 and E7 oncoproteins in HPV-induced anogenital malignancies. *Semin Virol* **7**: 295–304
Kurvinen K, Syrjänen K and Syrjänen, S (1996) p53 and bcl-2 proteins as prognostic markers in human papillomavirus-associated cervical lesions. *J Clin Onc* **14**: 2120–2130
Larson AA, Kern S, Sommers RL, Yokota J, Cavenee WK and Hampton GM (1996) Analysis of replication error phenotypes in cervical carcinoma. *Cancer Res* **56**: 1426–1431
Larson AA, Kern S, Curtiss S, Gordon R, Cavanee WK and Hampton GM (1997) High resolution analysis of chromosome 3p alterations in cervical cancer. *Cancer Res* **57**: 4082–4090
Lazo PA (1988*a*) Human papillomaviruses in oncogenesis. *BioEssays* **9**: 158–162
Lazo PA (1988*b*) Rearrangement of both alleles of human chromosome 8 in HeLa cells, one of them as a result of papillomavirus DNA integration. *J Biol Chem* **263**: 360–367
Lazo PA, Gallego MI, Ballester S and Feduchi E (1992) Genetic alterations by human papillomaviruses in oncogenesis. *FEBS Lett* **300**: 109–113
Le Beau MM, Drabkin H, Glover TW, Gemmill R, Rassool FV, McKeithan TW and Smith DI (1998) An FHIT tumor suppressor gene? *Genes Chromosom Cancer* **21**: 281–289
Leis PF, Stevens KR, Baer SC, Kadmon D, Goldberg LH and Wang XJ (1998) A c-rasHa mutation in the metastasis of a human papillomavirus (HPV)-18 positive penile squamous cell carcinoma suggests a cooperative effect between HPV-18 and c-rasHa activation in malignant progression. *Cancer* **83**: 122–129
Lengauer C, Kinzler KW and Vogelstein B (1998) Genetic instabilities in human cancers. *Nature* **396**: 643–649
Lopez-Borges S, Gallego MI and Lazo PA (1998) Recurrent integration of papillomavirus DNA within the human 12q14–15 uterine breakpoint region in genital carcinomas [In Process Citation]. *Genes Chromosomes Cancer* **23**: 55–60
Mao L, Fan Y-H, Lotan R and Hong WK (1996) Frequent abnormalities of FHIT, a candidate tumor suppressor gene, in head and neck cancer cell lines. *Cancer Res* **56**: 5128–5131
Mitelman F, Mertens F and Johansson B (1997) A breakpoint map of recurrent chromosomal rearrangements in human neoplasia. *Nat Genet* **15**: 417–474
Mitra AB, Murty V, Li RG, Pratap M, Luthra UK and Chaganti RSK (1994*a*). Allelotype analysis of cervical carcinoma. *Cancer Res* **54**: 4481–4487
Mitra AB, Murty V, Pratap M, Dodhani P and Chaganti RSK (1994*b*) ERBB2 (HER2/neu) oncogene is frequently amplified in squamous carcinoma of the uterine cervix. *Cancer Res* **54**: 637–639
Montgomery K, Tedford KL and McDougall JK (1995) Genetic instability of chromosome 3 in HPV immortalized and tumorigenic human keratinocytes. *Genes Chrom Cancer* **14**: 97–105
Muller CY, O'Boyle JD, Fong KM, Wistuba II, Biesterveld E, Ahmadian M, Miller DS, Gazdar AF and Minna JD (1998) Abnormalities of fragile histidine triad genomic and complementary DNA in cervical cancer: association with human papillomavirus type. *J Natl Cancer Inst* **90**: 433–439
Mullokandov MR, Kholodilov NG, Atkin NB, Burk RD, Johnson AB and Klinger HP (1996) Genomic alterations in cervical carcinoma: losses of chromosome heterozygosity and human papillomavirus tumor status. *Cancer Res* **56**: 197–205
Ning Y, Weber JL, Killary AM, Ledbetter DH, Smith JR and Pereira-Smith OM (1991) Genetic analysis of indefinite division in human cells: evidence for a cell senescence-related gene(s) on human chromosome 4. *Proc Natl Acad Sci USA* **88**: 5635–5639
O'Connor MJ, Tan S, Tan C and Bernard H (1996) YY1 represses human papillomavirus type 16 transcription by quenching AP-1 activity *J Virol* **70**: 6529–6539
Oshimura M, Kugoh H, Koi M, Shimizu M, Yamada H, Satoh H and Barrett JC (1990) Transfer of a normal human chromosome 11 suppresses tumorigenicity of some but not all tumor cell lines. *J Cell Biochem* **42**: 135–142
Park S, Kang Y, Kim B, Lee S, Lee E, Lee K, Park K and Lee J (1995) Loss of heterozygosity on the short arm of chromosome 17 in uterine cervical carcinomas. *Cancer Genet Cytogenet* **79**: 74–78
Pinion SB, Kennedy JH, Miller RW and MacLean AB (1991) Oncogene expression in cervical intraepithelial neoplasia and invasive cancer of the cervix. *Lancet* **337**: 819–820
Pirisi L, Yasumoto S, Feller M, Doniger J and DiPaolo JA (1987) Transformation of human fibroblasts and keratinocytes with human papillomavirus type 16 DNA. *J Virol* **61**: 1061–1066
Popescu NC and DiPaolo JA (1989) Preferential sites for viral integration on mammalian genome. *Cancer Genet Cytogenet* **42**: 157–171
Popescu NC, DiPaolo JA and Amsbaugh SC (1987) Integration sites of human papillomavirus 18 DNA sequences on HeLa cell chromosomes. *Cytogenet Cell Genet* **44**: 58–62
Popescu NC, Zimonjic D and DiPaolo JA (1990) Viral integration, fragile sites and proto-oncogenes in human neoplasia. *Hum Genet* **44**: 58–62
Rabbitts TH (1994) Chromosomal translocations in human cancer. *Nature* **372**: 143–149.
Rabbitts TH (1997) Chromosomal breakpoints hit the spot. *Nat Med* **3**: 496–497

© 1999 Cancer Research Campaign 

Rosl F, Achtstatter T, Bauknecht T, Hutter KJ, Futterman G and zur Hausen H (1991) Extinction of the HPV18 upstream regulatory region in cervical carcinoma cells after fusion with non-tumorigenic human keratinocytes under non-selective conditions. *EMBO J* **10**: 1337–1345

Sánchez-García I (1997) Consequences of chromosomal abnormalities in tumor development. *Annu Rev Genet* **31**: 429–453

Sastre-Garau X, Couturier J, Favre M and Orth G (1995) A recurrent human papillomavirus integration site at chromosome region 12q14–15 in SW756 and SK-v cell lines derived from genital tumors. *CR Acad Sci III* **318**: 475–478

Saxon PJ, Srivatsan ES and Stanbridge EJ (1986) Introduction of human chromosome 11 via microcell transfer controls tumorigenic expression of HeLa cells. *Embo J* **5**: 3461–3466

Schneider JF, McGlennen RC, LaBresh KV, Ostrow RS and Faras AJ (1991) Rhesus papillomavirus type 1 cooperates with activated ras in transforming primary epithelial rat cells independent of dexamethasone. *J Virol* **65**: 3354–3358

Shah KV and Howley PM (1995) Papillomaviruses. In *Virology*, Fields BN, Knipe DM & Howley PN (eds), Vol. 2. pp. 2077–2109. Lippincot-Raven: Philadelphia

Smith PP, Friedman CL, Bryant EM and McDougall JK (1992) Viral integration and fragile sites in human papillomavirus immortalized human keratinocyte cell lines. *Genes Chromosomes Cancer* **5**: 150–157

Southern SA and Herrington CS (1997) Interphase karyotypic analysis of chromosomes 11, 17 and X in invasive squamous-cell carcinoma pf the cervix: Morphological correlation with HPV infection. *Int J Cancer* **70**: 502–507

Sozzi G, Veronese ML, Negrini M, Baffa R, Cotticelli MG, Inoue H, Tornielli, S, Pilotti S, De Gregorio L, Pastorino U, Pierotti MA, Ohta M, Huebner K and Croce CM (1996) The FHIT gene at 3p14.2 is abnormal in lung cancer. *Cell* **85**: 17–26

Srivasan ES, Misra BC, Venugopalan M and Wilczynski SP (1991) Loss of heterozygosity for alleles on chromosome 11 in cervical carcinoma. *Am J Hum Genet* **49**: 868–877

Steenbergen RDM, Hermsen M, Walboomers JMM, Joenje H, Arwert F, Meijer C and Snijders PJF (1995) Integrated human papillomavirus type 16 and loss of heterozygosity at 11q22 and 18q21 in an oral carcinoma and its derivative cell line. *Cancer Res* **55**: 5465–5471

Steenbergen RDM, Walboomers JMM, Meijer C, van der Raaij-Helmer EMH, Parker JN, Chow LT, Broker TR and Snijders PJF (1996) Transition of human papillomavirus type 16 and 18 transfected human foreskin keratinocytes towards immortality: activation of telomerase and allele loss at 3p, 10p, 11q and/or 18q. *Oncogene* **13**: 1249–1257

Stoppler H, Stoppler MC, Johnson E, Simbulan-Rosenthal CM, Smulson ME, lyer S, Rosenthal DS and Schlegel R (1998) The E7 protein of human papillomavirus type 16 sensitizes primary human keratinocytes to apoptosis. *Oncogene* **17**: 1207–1214

Thiagalingam S, Lisistsyn NA, Hamaguchi M, Wigler MH, Willson JKV, Markowitz SD, Leach FS, Kinzler KW and Vogelstein B (1996) Evaluation of the FHIT gene in colorectal cancers. *Cancer Res* **56**: 2936–2939

Thomas M, Matlashewski G, Pim D and Banks L (1996) Induction of apoptosis by p53 is independent of its oligomeric state and can be abolished by HPV18 E6 through ubiquitin mediated degradation. *Oncogene* **13**: 265–273

Tomlinson I and Bodmer W (1999) Selection, the mutation rate and cancer: ensuring that the tail does not wag the tail. *Nat Med* **5**: 11–12

Tomlinson IPM, Novelli MR and Bodmer WF (1996) The mutation rate and cancer. *Proc Natl Acad Sci USA* **93**: 14800–14803

Uejima H, Mitsuya K, Kugoh H, Horikawa I and Oshimura M (1995) Normal human chromosome 2 induces cellular senescence in the human cervical carcinoma cell line SiHa. *Genes Chromosomes Cancer* **14**: 120–127

Unger ER, Vernon SD, Thoms WW, Nisenbaum R, Spann CO, Horowitz IR, Icenogle JP and Reeves WC (1995) Human papillomavirus and disease-free survival in FIGO stage Ib cervical cancer. *J Infect Dis* **172**: 1184–1190

Vernon SD, Unger ER, Miller DL, Lee DR and Reeves WC (1997) Association of human papillomavirus type 16 integration in the E2 gene with poor disease-free survival from cervical cancer. *Int J Cancer* **74**: 50–56

Werness BA, Levine AJ and Howley PM (1990) Association of human papillomavirus types 16 and 18 E6 proteins with p53. *Science* **248**: 76–79

Wilke CM, Hall BK, Hoge A, Paradee W, Smith DI and Glover TW (1996) FRA3B extends over a broad region and contains a spontaneous HPV16 integration site: direct evidence for the coincidence of viral integration sites and fragile sites. *Hum Mol Genet* **5**: 187–195

Yokota J, Tsukada Y, Nakajima T, Gotoh M, Shimosato Y, Mori N, Tsunokawa Y, Sugimura T and Terada M (1989) Loss of heterozygosity on the short arm of chromosome 3 in carcinoma of the uterine cervix. *Cancer Res* **49**: 3598–3601

Yoshino K, Enomoto T, Nakamura T, Nakashima R, Wada H, Saitoh J, Noda K and Murata Y (1998) Aberrant FHIT transcripts in squamous cell carcinoma of the uterine cervix. *Int J Cancer* **76**: 176–181

Zimonjic DB, Popescu NC and DiPaolo JA (1994) Chromosomal organization of viral integration sites in human papillomavirus-immortalized human keratynocytes cell lines. *Cancer Genet Cytogenet* **72**: 39–43

Zimonjic DB, Druck T, Ohta M, Kastury K, Coce CM, Popescu N and Huebner K (1997) Position of chromosome 3p14.2 fragile site (FRA3B) within the FHIT gene. *Cancer Res* **57**: 1166–1170

Zou TT, Lei J, Shi YQ, Wang S, Souza RF, Kong D, Shimada Y, Smolinski KN, Greenwald BD, Abraham JM, Harpaz N and Meltzer SJ (1997) FHIT alterations in esophageal cancer and ulcerative colitis (UC). *Oncogene* **15**: 101–105

zur Hausen H (1994) Human papillomaviruses. *Annu Rev Microbiol* **48**: 427–447

 *© 1999 Cancer Research Campaign*

## References

[PDF_00831] Antinore M. J., Birrer M. J., Patel D., Nader L., McCance D. J. (1996). The human papillomavirus type 16 E7 gene product interacts with and trans-activates the AP1 family of transcription factors.. EMBO J.

[PDF_00834] Atkin N. B., Baker M. C., Fox M. F. (1990). Chromosome changes in 43 carcinomas of the cervix uteri.. Cancer Genet Cytogenet.

[PDF_00836] Banks L., Edmonds C., Vousden K. H. (1990). Ability of the HPV16 E7 protein to bind RB and induce DNA synthesis is not sufficient for efficient transforming activity in NIH3T3 cells.. Oncogene.

[PDF_00839] Bartholomew J. S., Glenville S., Sarkar S., Burt D. J., Stanley M. A., Ruiz-Cabello F., Chengang J., Garrido F., Stern P. L. (1997). Integration of high-risk human papillomavirus DNA is linked to the down-regulation of class I human leukocyte antigens by steroid hormones in cervical tumor cells.. Cancer Res.

[PDF_00843] Bartsch D., Boye B., Baust C., zur Hausen H., Schwarz E. (1992). Retinoic acid-mediated repression of human papillomavirus 18 transcription and different ligand regulation of the retinoic acid receptor beta gene in non-tumorigenic and tumorigenic HeLa hybrid cells.. EMBO J.

[PDF_00847] Bauer-Hofmann R., Borghouts C., Auvinen E., Bourda E., Rösl F., Alonso A. (1996). Genomic cloning and characterization of the nonoccupied allele corresponding to the integration site of human papillomavirus type 16 DNA in the cervical cancer cell line SiHa.. Virology.

[PDF_00851] Bethwaite P. B., Koreth J., Herrington C. S., McGee J. O. (1995). Loss of heterozygosity occurs at the D11S29 locus on chromosome 11q23 in invasive cervical carcinoma.. Br J Cancer.

[PDF_00853] Bosch F. X., Manos M. M., Muñoz N., Sherman M., Jansen A. M., Peto J., Schiffman M. H., Moreno V., Kurman R., Shah K. V. (1995). Prevalence of human papillomavirus in cervical cancer: a worldwide perspective. International biological study on cervical cancer (IBSCC) Study Group.. J Natl Cancer Inst.

[PDF_00857] Braly P. (1996). Preventing cervical cancer.. Nat Med.

[PDF_00858] Cannistra S. A., Niloff J. M. (1996). Cancer of the uterine cervix.. N Engl J Med.

[PDF_00860] Castrén K., Vähäkangas K., Heikkinen E., Ranki A. (1998). Absence of p53 mutations in benign and pre-malignant male genital lesions with over-expressed p53 protein.. Int J Cancer.

[PDF_00869] Chen T. M., Pecoraro G., Defendi V. (1993). Genetic analysis of in vitro progression of human papillomavirus-transfected human cervical cells.. Cancer Res.

[PDF_00875] Choo K. B., Huang C. J., Chen C. M., Han C. P., Au L. C. (1995). Jun-B oncogene aberrations in cervical cancer cell lines.. Cancer Lett.

[PDF_00872] Choo K. B., Lee H. H., Liew L. N., Chong K. Y., Chou H. F. (1990). Analysis of the unoccupied site of an integrated human papillomavirus 16 sequence in a cervical carcinoma.. Virology.

[PDF_00877] Chu T. Y., Shen C. Y., Chiou Y. S., Lu J. J., Perng C. L., Yu M. S., Liu H. S. (1998). HPV-associated cervical cancers show frequent allelic loss at 3p14 but no apparent aberration of FHIT mRNA.. Int J Cancer.

[PDF_00863] Couturier J., Sastre-Garau X., Schneider-Maunoury S., Labib A., Orth G. (1991). Integration of papillomavirus DNA near myc genes in genital carcinomas and its consequences for proto-oncogene expression.. J Virol.

[PDF_00866] Cullen A. P., Reid R., Campion M., Lörincz A. T. (1991). Analysis of the physical state of different human papillomavirus DNAs in intraepithelial and invasive cervical neoplasm.. J Virol.

[PDF_00880] Dokianakis D. N., Sourvinos G., Sakkas S., Athanasiadou E., Spandidos D. A. (1998). Detection of HPV and ras gene mutations in cervical smears from female genital lesions.. Oncol Rep.

[PDF_00883] Dowhanick J. J., McBride A. A., Howley P. M. (1995). Suppression of cellular proliferation by the papillomavirus E2 protein.. J Virol.

[PDF_00885] Dürst M., Croce C. M., Gissmann L., Schwarz E., Huebner K. (1987). Papillomavirus sequences integrate near cellular oncogenes in some cervical carcinomas.. Proc Natl Acad Sci U S A.

[PDF_00891] Frattini M. G., Hurst S. D., Lim H. B., Swaminathan S., Laimins L. A. (1997). Abrogation of a mitotic checkpoint by E2 proteins from oncogenic human papillomaviruses correlates with increased turnover of the p53 tumor suppressor protein.. EMBO J.

[PDF_00895] Fujita M., Inoue M., Tanizawa O., Iwamoto S., Enomoto T. (1992). Alterations of the p53 gene in human primary cervical carcinoma with and without human papillomavirus infection.. Cancer Res.

[PDF_00898] Gallego M. I., Lazo P. A. (1995). Deletion in human chromosome region 12q13-15 by integration of human papillomavirus DNA in a cervical carcinoma cell line.. J Biol Chem.

[PDF_00901] Gallego M. I., Schoenmakers E. F., Van de Ven W. J., Lazo P. A. (1997). Complex genomic rearrangement within the 12q15 multiple aberration region induced by integrated human papillomavirus 18 in a cervical carcinoma cell line.. Mol Carcinog.

[PDF_00905] Gloss B., Chong T., Bernard H. U. (1989). Numerous nuclear proteins bind the long control region of human papillomavirus type 16: a subset of 6 of 23 DNase I-protected segments coincides with the location of the cell-type-specific enhancer.. J Virol.

[PDF_00909] Greenblatt M. S., Bennett W. P., Hollstein M., Harris C. C. (1994). Mutations in the p53 tumor suppressor gene: clues to cancer etiology and molecular pathogenesis.. Cancer Res.

[PDF_00912] Greenspan D. L., Connolly D. C., Wu R., Lei R. Y., Vogelstein J. T., Kim Y. T., Mok J. E., Muñoz N., Bosch F. X., Shah K. (1997). Loss of FHIT expression in cervical carcinoma cell lines and primary tumors.. Cancer Res.

[PDF_00916] Hampton G. M., Larson A. A., Baergen R. N., Sommers R. L., Kern S., Cavenee W. K. (1996). Simultaneous assessment of loss of heterozygosity at multiple microsatellite loci using semi-automated fluorescence-based detection: subregional mapping of chromosome 4 in cervical carcinoma.. Proc Natl Acad Sci U S A.

[PDF_00921] Hampton G. M., Penny L. A., Baergen R. N., Larson A., Brewer C., Liao S., Busby-Earle R. M., Williams A. W., Steel C. M., Bird C. C. (1994). Loss of heterozygosity in cervical carcinoma: subchromosomal localization of a putative tumor-suppressor gene to chromosome 11q22-q24.. Proc Natl Acad Sci U S A.

[PDF_00926] Hayashi S., Tanimoto K., Hajiro-Nakanishi K., Tsuchiya E., Kurosumi M., Higashi Y., Imai K., Suga K., Nakachi K. (1997). Abnormal FHIT transcripts in human breast carcinomas: a clinicopathological and epidemiological analysis of 61 Japanese cases.. Cancer Res.

[PDF_00930] Helland A., Børresen-Dale A. L., Peltomäki P., Hektoen M., Kristensen G. B., Nesland J. M., de la Chapelle A., Lothe R. A. (1997). Microsatellite instability in cervical and endometrial carcinomas.. Int J Cancer.

[PDF_00933] Helland A., Karlsen F., Due E. U., Holm R., Kristensen G., Børresen-Dale A. l. (1998). Mutations in the TP53 gene and protein expression of p53, MDM 2 and p21/WAF-1 in primary cervical carcinomas with no or low human papillomavirus load.. Br J Cancer.

[PDF_00937] Helland A., Olsen A. O., Gjøen K., Akselsen H. E., Sauer T., Magnus P., Børresen-Dale A. L., Rønningen K. S. (1998). An increased risk of cervical intra-epithelial neoplasia grade II-III among human papillomavirus positive patients with the HLA-DQA1*0102-DQB1*0602 haplotype: a population-based case-control study of Norwegian women.. Int J Cancer.

[PDF_00947] Heselmeyer K., Macville M., Schröck E., Blegen H., Hellström A. C., Shah K., Auer G., Ried T. (1997). Advanced-stage cervical carcinomas are defined by a recurrent pattern of chromosomal aberrations revealing high genetic instability and a consistent gain of chromosome arm 3q.. Genes Chromosomes Cancer.

[PDF_00943] Heselmeyer K., Schröck E., du Manoir S., Blegen H., Shah K., Steinbeck R., Auer G., Ried T. (1996). Gain of chromosome 3q defines the transition from severe dysplasia to invasive carcinoma of the uterine cervix.. Proc Natl Acad Sci U S A.

[PDF_00955] Hueber A. O., Zörnig M., Lyon D., Suda T., Nagata S., Evan G. I. (1997). Requirement for the CD95 receptor-ligand pathway in c-Myc-induced apoptosis.. Science.

[PDF_00958] Huettner P. C., Gerhard D. S., Li L., Gersell D. J., Dunnigan K., Kamarasova T., Rader J. S. (1998). Loss of heterozygosity in clinical stage IB cervical carcinoma: relationship with clinical and histopathologic features.. Hum Pathol.

[PDF_00962] Iglesias M., Yen K., Gaiotti D., Hildesheim A., Stoler M. H., Woodworth C. D. (1998). Human papillomavirus type 16 E7 protein sensitizes cervical keratinocytes to apoptosis and release of interleukin-1alpha.. Oncogene.

[PDF_00969] Jeon S., Allen-Hoffmann B. L., Lambert P. F. (1995). Integration of human papillomavirus type 16 into the human genome correlates with a selective growth advantage of cells.. J Virol.

[PDF_00965] Jeon S., Lambert P. F. (1995). Integration of human papillomavirus type 16 DNA into the human genome leads to increased stability of E6 and E7 mRNAs: implications for cervical carcinogenesis.. Proc Natl Acad Sci U S A.

[PDF_00972] Jones M. H., Nakamura Y. (1992). Deletion mapping of chromosome 3p in female genital tract malignancies using microsatellite polymorphisms.. Oncogene.

[PDF_00975] Kaelbling M., Burk R. D., Atkin N. B., Johnson A. B., Klinger H. P. (1992). Loss of heterozygosity on chromosome 17p and mutant p53 in HPV-negative cervical carcinomas.. Lancet.

[PDF_00978] Kalantari M., Karlsen F., Kristensen G., Holm R., Hagmar B., Johansson B. (1998). Disruption of the E1 and E2 reading frames of HPV 16 in cervical carcinoma is associated with poor prognosis.. Int J Gynecol Pathol.

[PDF_00981] Kamijo T., Zindy F., Roussel M. F., Quelle D. E., Downing J. R., Ashmun R. A., Grosveld G., Sherr C. J. (1997). Tumor suppression at the mouse INK4a locus mediated by the alternative reading frame product p19ARF.. Cell.

[PDF_00984] Karlsen F., Rabbitts P. H., Sundresan V., Hagmar B. (1994). PCR-RFLP studies on chromosome 3p in formaldehyde-fixed, paraffin-embedded cervical cancer tissues.. Int J Cancer.

[PDF_00987] Kersemaekers A. M., Hermans J., Fleuren G. J., van de Vijver M. J. (1998). Loss of heterozygosity for defined regions on chromosomes 3, 11 and 17 in carcinomas of the uterine cervix.. Br J Cancer.

[PDF_00990] Kersemaekers A. M., Kenter G. G., Hermans J., Fleuren G. J., van de Vijver M. J. (1998). Allelic loss and prognosis in carcinoma of the uterine cervix.. Int J Cancer.

[PDF_00993] Kim J. W., Namkoong S. E., Ryu S. W., Kim H. S., Shin J. W., Lee J. M., Kim D. H., Kim I. K. (1998). Absence of p15INK4B and p16INK4A gene alterations in primary cervical carcinoma tissues and cell lines with human papillomavirus infection.. Gynecol Oncol.

[PDF_00999] Kinzler K. W., Vogelstein B. (1998). Landscaping the cancer terrain.. Science.

[PDF_00997] Kinzler K. W., Vogelstein B. (1996). Lessons from hereditary colorectal cancer.. Cell.

[PDF_01001] Kirchhoff M., Rose H., Petersen B. L., Maahr J., Gerdes T., Lundsteen C., Bryndorf T., Kryger-Baggesen N., Christensen L., Engelholm S. A. (1999). Comparative genomic hybridization reveals a recurrent pattern of chromosomal aberrations in severe dysplasia/carcinoma in situ of the cervix and in advanced-stage cervical carcinoma.. Genes Chromosomes Cancer.

[PDF_01006] Kohno T., Takayama H., Hamaguchi M., Takano H., Yamaguchi N., Tsuda H., Hirohashi S., Vissing H., Shimizu M., Oshimura M. (1993). Deletion mapping of chromosome 3p in human uterine cervical cancer.. Oncogene.

[PDF_01009] Koi M., Morita H., Yamada H., Satoh H., Barrett J. C., Oshimura M. (1989). Normal human chromosome 11 suppresses tumorigenicity of human cervical tumor cell line SiHa.. Mol Carcinog.

[PDF_01012] Konishi H., Takahashi T., Kozaki K., Yatabe Y., Mitsudomi T., Fujii Y., Sugiura T., Matsuda H., Takahashi T., Takahashi T. (1998). Detailed deletion mapping suggests the involvement of a tumor suppressor gene at 17p13.3, distal to p53, in the pathogenesis of lung cancers.. Oncogene.

[PDF_01016] Krammer P. H. (1997). The tumor strikes back: new data on expression of the CD95(APO-1/Fas) receptor/ligand system may cause paradigm changes in our view on drug treatment and tumor immunology.. Cell Death Differ.

[PDF_01019] Ku J. L., Kim W. H., Park H. S., Kang S. B., Park J. G. (1997). Establishment and characterization of 12 uterine cervical-carcinoma cell lines: common sequence variation in the E7 gene of HPV-16-positive cell lines.. Int J Cancer.

[PDF_01023] Ku W. H., Liu I. L., Yen M. S., Chang Chien C. C., Yue C. T., Ma Y. Y., Chang S. F., Ng H. T., Wu C. W., Shen C. Y. (1997). Genomic deletion and p53 inactivation in cervical carcinoma.. Int J Cancer.

[PDF_01028] Kurvinen K., Syrjänen K., Syrjänen S. (1996). p53 and bcl-2 proteins as prognostic markers in human papillomavirus-associated cervical lesions.. J Clin Oncol.

[PDF_01034] Larson A. A., Kern S., Curtiss S., Gordon R., Cavenee W. K., Hampton G. M. (1997). High resolution analysis of chromosome 3p alterations in cervical carcinoma.. Cancer Res.

[PDF_01031] Larson A. A., Kern S., Sommers R. L., Yokota J., Cavenee W. K., Hampton G. M. (1996). Analysis of replication error (RER+) phenotypes in cervical carcinoma.. Cancer Res.

[PDF_01041] Lazo P. A., Gallego M. I., Ballester S., Feduchi E. (1992). Genetic alterations by human papillomaviruses in oncogenesis.. FEBS Lett.

[PDF_01037] Lazo P. A. (1988). Human papillomaviruses in oncogenesis.. Bioessays.

[PDF_01038] Lazo P. A. (1988). Rearrangement of both alleles of human chromosome 8 in HeLa cells, one of them as a result of papillomavirus DNA integration.. J Biol Chem.

[PDF_01043] Le Beau M. M., Drabkin H., Glover T. W., Gemmill R., Rassool F. V., McKeithan T. W., Smith D. I. (1998). An FHIT tumor suppressor gene?. Genes Chromosomes Cancer.

[PDF_01046] Leis P. F., Stevens K. R., Baer S. C., Kadmon D., Goldberg L. H., Wang X. J. (1998). A c-rasHa mutation in the metastasis of a human papillomavirus (HPV)-18 positive penile squamous cell carcinoma suggests a cooperative effect between HPV-18 and c-rasHa activation in malignant progression.. Cancer.

[PDF_01051] Lengauer C., Kinzler K. W., Vogelstein B. (1998). Genetic instabilities in human cancers.. Nature.

[PDF_01053] Lopez-Borges S., Gallego M. I., Lazo P. A. (1998). Recurrent integration of papillomavirus DNA within the human 12q14-15 uterine breakpoint region in genital carcinomas.. Genes Chromosomes Cancer.

[PDF_01057] Mao L., Fan Y. H., Lotan R., Hong W. K. (1996). Frequent abnormalities of FHIT, a candidate tumor suppressor gene, in head and neck cancer cell lines.. Cancer Res.

[PDF_01060] Mitelman F., Mertens F., Johansson B. (1997). A breakpoint map of recurrent chromosomal rearrangements in human neoplasia.. Nat Genet.

[PDF_01062] Mitra A. B., Murty V. V., Li R. G., Pratap M., Luthra U. K., Chaganti R. S. (1994). Allelotype analysis of cervical carcinoma.. Cancer Res.

[PDF_01064] Mitra A. B., Murty V. V., Pratap M., Sodhani P., Chaganti R. S. (1994). ERBB2 (HER2/neu) oncogene is frequently amplified in squamous cell carcinoma of the uterine cervix.. Cancer Res.

[PDF_01067] Montgomery K. D., Tedford K. L., McDougall J. K. (1995). Genetic instability of chromosome 3 in HPV-immortalized and tumorigenic human keratinocytes.. Genes Chromosomes Cancer.

[PDF_01070] Muller C. Y., O'Boyle J. D., Fong K. M., Wistuba I. I., Biesterveld E., Ahmadian M., Miller D. S., Gazdar A. F., Minna J. D. (1998). Abnormalities of fragile histidine triad genomic and complementary DNAs in cervical cancer: association with human papillomavirus type.. J Natl Cancer Inst.

[PDF_01074] Mullokandov M. R., Kholodilov N. G., Atkin N. B., Burk R. D., Johnson A. B., Klinger H. P. (1996). Genomic alterations in cervical carcinoma: losses of chromosome heterozygosity and human papilloma virus tumor status.. Cancer Res.

[PDF_01078] Ning Y., Weber J. L., Killary A. M., Ledbetter D. H., Smith J. R., Pereira-Smith O. M. (1991). Genetic analysis of indefinite division in human cells: evidence for a cell senescence-related gene(s) on human chromosome 4.. Proc Natl Acad Sci U S A.

[PDF_01082] O'Connor M. J., Tan S. H., Tan C. H., Bernard H. U. (1996). YY1 represses human papillomavirus type 16 transcription by quenching AP-1 activity.. J Virol.

[PDF_01085] Oshimura M., Kugoh H., Koi M., Shimizu M., Yamada H., Satoh H., Barrett J. C. (1990). Transfer of a normal human chromosome 11 suppresses tumorigenicity of some but not all tumor cell lines.. J Cell Biochem.

[PDF_01088] Park S. Y., Kang Y. S., Kim B. G., Lee S. H., Lee E. D., Lee K. H., Park K. B., Lee J. H. (1995). Loss of heterozygosity on the short arm of chromosome 17 in uterine cervical carcinomas.. Cancer Genet Cytogenet.

[PDF_01091] Pinion S. B., Kennedy J. H., Miller R. W., MacLean A. B. (1991). Oncogene expression in cervical intraepithelial neoplasia and invasive cancer of cervix.. Lancet.

[PDF_01094] Pirisi L., Yasumoto S., Feller M., Doniger J., DiPaolo J. A. (1987). Transformation of human fibroblasts and keratinocytes with human papillomavirus type 16 DNA.. J Virol.

[PDF_01099] Popescu N. C., DiPaolo J. A., Amsbaugh S. C. (1987). Integration sites of human papillomavirus 18 DNA sequences on HeLa cell chromosomes.. Cytogenet Cell Genet.

[PDF_01097] Popescu N. C., DiPaolo J. A. (1989). Preferential sites for viral integration on mammalian genome.. Cancer Genet Cytogenet.

[PDF_01106] Rabbitts T. H. (1997). Chromosomal breakpoints hit the spot.. Nat Med.

[PDF_01104] Rabbitts T. H. (1994). Chromosomal translocations in human cancer.. Nature.

[PDF_01112] Rösl F., Achtstätter T., Bauknecht T., Hutter K. J., Futterman G., zur Hausen H. (1991). Extinction of the HPV18 upstream regulatory region in cervical carcinoma cells after fusion with non-tumorigenic human keratinocytes under non-selective conditions.. EMBO J.

[PDF_01118] Sastre-Garau X., Couturier J., Favre M., Orth G. (1995). A recurrent human papillomavirus integration site at chromosome region 12q14-q15 in SW756 and SK-v cell lines derived from genital tumors.. C R Acad Sci III.

[PDF_01122] Saxon P. J., Srivatsan E. S., Stanbridge E. J. (1986). Introduction of human chromosome 11 via microcell transfer controls tumorigenic expression of HeLa cells.. EMBO J.

[PDF_01125] Schneider J. F., McGlennen R. C., LaBresh K. V., Ostrow R. S., Faras A. J. (1991). Rhesus papillomavirus type 1 cooperates with activated ras in transforming primary epithelial rat cells independent of dexamethasone.. J Virol.

[PDF_01130] Smith P. P., Friedman C. L., Bryant E. M., McDougall J. K. (1992). Viral integration and fragile sites in human papillomavirus-immortalized human keratinocyte cell lines.. Genes Chromosomes Cancer.

[PDF_01133] Southern S. A., Herrington C. S. (1997). Interphase karyotypic analysis of chromosomes 11, 17 and X in invasive squamous-cell carcinoma of the cervix: morphological correlation with HPV infection.. Int J Cancer.

[PDF_01136] Sozzi G., Veronese M. L., Negrini M., Baffa R., Cotticelli M. G., Inoue H., Tornielli S., Pilotti S., De Gregorio L., Pastorino U. (1996). The FHIT gene 3p14.2 is abnormal in lung cancer.. Cell.

[PDF_01140] Srivatsan E. S., Misra B. C., Venugopalan M., Wilczynski S. P. (1991). Loss of heterozygosity for alleles on chromosome II in cervical carcinoma.. Am J Hum Genet.

[PDF_01143] Steenbergen R. D., Hermsen M. A., Walboomers J. M., Joenje H., Arwert F., Meijer C. J., Snijders P. J. (1995). Integrated human papillomavirus type 16 and loss of heterozygosity at 11q22 and 18q21 in an oral carcinoma and its derivative cell line.. Cancer Res.

[PDF_01147] Steenbergen R. D., Walboomers J. M., Meijer C. J., van der Raaij-Helmer E. M., Parker J. N., Chow L. T., Broker T. R., Snijders P. J. (1996). Transition of human papillomavirus type 16 and 18 transfected human foreskin keratinocytes towards immortality: activation of telomerase and allele losses at 3p, 10p, 11q and/or 18q.. Oncogene.

[PDF_01152] Stöppler H., Stöppler M. C., Johnson E., Simbulan-Rosenthal C. M., Smulson M. E., Iyer S., Rosenthal D. S., Schlegel R. (1998). The E7 protein of human papillomavirus type 16 sensitizes primary human keratinocytes to apoptosis.. Oncogene.

[PDF_01116] Sánchez-García I. (1997). Consequences of chromosomal abnormalities in tumor development.. Annu Rev Genet.

[PDF_01156] Thiagalingam S., Lisitsyn N. A., Hamaguchi M., Wigler M. H., Willson J. K., Markowitz S. D., Leach F. S., Kinzler K. W., Vogelstein B. (1996). Evaluation of the FHIT gene in colorectal cancers.. Cancer Res.

[PDF_01159] Thomas M., Matlashewski G., Pim D., Banks L. (1996). Induction of apoptosis by p53 is independent of its oligomeric state and can be abolished by HPV-18 E6 through ubiquitin mediated degradation.. Oncogene.

[PDF_01164] Tomlinson I. P., Novelli M. R., Bodmer W. F. (1996). The mutation rate and cancer.. Proc Natl Acad Sci U S A.

[PDF_01162] Tomlinson I., Bodmer W. (1999). Selection, the mutation rate and cancer: ensuring that the tail does not wag the dog.. Nat Med.

[PDF_01166] Uejima H., Mitsuya K., Kugoh H., Horikawa I., Oshimura M. (1995). Normal human chromosome 2 induces cellular senescence in the human cervical carcinoma cell line SiHa.. Genes Chromosomes Cancer.

[PDF_01169] Unger E. R., Vernon S. D., Thoms W. W., Nisenbaum R., Spann C. O., Horowitz I. R., Icenogle J. P., Reeves W. C. (1995). Human papillomavirus and disease-free survival in FIGO stage Ib cervical cancer.. J Infect Dis.

[PDF_01172] Vernon S. D., Unger E. R., Miller D. L., Lee D. R., Reeves W. C. (1997). Association of human papillomavirus type 16 integration in the E2 gene with poor disease-free survival from cervical cancer.. Int J Cancer.

[PDF_01175] Werness B. A., Levine A. J., Howley P. M. (1990). Association of human papillomavirus types 16 and 18 E6 proteins with p53.. Science.

[PDF_01178] Wilke C. M., Hall B. K., Hoge A., Paradee W., Smith D. I., Glover T. W. (1996). FRA3B extends over a broad region and contains a spontaneous HPV16 integration site: direct evidence for the coincidence of viral integration sites and fragile sites.. Hum Mol Genet.

[PDF_01182] Yokota J., Tsukada Y., Nakajima T., Gotoh M., Shimosato Y., Mori N., Tsunokawa Y., Sugimura T., Terada M. (1989). Loss of heterozygosity on the short arm of chromosome 3 in carcinoma of the uterine cervix.. Cancer Res.

[PDF_01186] Yoshino K., Enomoto T., Nakamura T., Nakashima R., Wada H., Saitoh J., Noda K., Murata Y. (1998). Aberrant FHIT transcripts in squamous cell carcinoma of the uterine cervix.. Int J Cancer.

[PDF_01192] Zimonjic D. B., Druck T., Ohta M., Kastury K., Croce C. M., Popescu N. C., Huebner K. (1997). Positions of chromosome 3p14.2 fragile sites (FRA3B) within the FHIT gene.. Cancer Res.

[PDF_01189] Zimonjic D. B., Popescu N. D., DiPaolo J. A. (1994). Chromosomal organization of viral integration sites in human papillomavirus-immortalized human keratinocyte cell lines.. Cancer Genet Cytogenet.

[PDF_01195] Zou T. T., Lei J., Shi Y. Q., Yin J., Wang S., Souza R. F., Kong D., Shimada Y., Smolinski K. N., Greenwald B. D. (1997). FHIT gene alterations in esophageal cancer and ulcerative colitis (UC).. Oncogene.

[PDF_00888] el Awady M. K., Kaplan J. B., O'Brien S. J., Burk R. D. (1987). Molecular analysis of integrated human papillomavirus 16 sequences in the cervical cancer cell line SiHa.. Virology.

[PDF_01199] zur Hausen H., de Villiers E. M. (1994). Human papillomaviruses.. Annu Rev Microbiol.

